# Efficient Direct Recycling of Spent Batteries: Integrated Lithiation and Delamination

**DOI:** 10.1002/advs.75879

**Published:** 2026-06-01

**Authors:** Jeonghwan Song, Seok Hyun Song, Dokyeong Han, Jaehyun Noh, Jayoung Kim, Min Jeong Kim, Hayong Song, Jeong‐Sun Park, Jaekook Kim, Jiyoung Ma, Jinju Song, Jung‐Je Woo

**Affiliations:** ^1^ Gwangju Clean Energy Research Center Korea Institute of Energy Research (KIER) Gwangju South Korea; ^2^ School of Materials Science and Engineering Chonnam National University (CNU) Gwangju South Korea; ^3^ Graduate School of Energy Convergence Gwangju Institute of Science and Technology (GIST) Gwangju South Korea; ^4^ Department of Materials Science and Engineering Gwangju Institute of Science and Technology (GIST) Gwangju South Korea

**Keywords:** delamination, direct recycling, integrated process, polyol, relithiation

## Abstract

Direct recycling offers a promising alternative for regenerating spent Li‐ion batteries (LIBs); however, achieving high‐purity cathode and ensuring cost‐effectiveness remains challenging. Herein, we present an innovative integrated process that simultaneously enables delamination and relithiation of spent cathodes, Li_x_Ni_0.6_Co_0.2_Mn_0.2_O_2_ and Li_x_FePO_4_, under open‐air conditions with minimal energy. By oxidizing diethylene glycol, a green solvent, the transition‐metal oxidation state in the cathode is effectively reduced, thereby facilitating Li reinsertion into the structure. Consequently, the formation of glycol aldehyde induces 100% electrode delamination, enabling a one‐pot restoration pathway. The reaction mechanism is elucidated using Proton nuclear magnetic resonance (^1^H‐NMR), Soft X‐ray absorption spectroscopy (soft‐XAS), and X‐ray absorption near‐edge structure spectroscopy (XANES), and the regenerated cathodes consistently recover over 99% of their electrochemical performance for both 2 and 50 Ah cells. Furthermore, the regeneration solution is reusable, requiring only Li salt supplementation. This study presents a practical, low‐cost, and environmentally friendly approach for advancing the scalability and feasibility of direct LIB recycling technologies.

## Graphical abstract

An integrated process for the delamination and relithiation of spent cathodes, Li_x_Ni_0.6_Co_0.2_Mn_0.2_O_2_ and Li_x_FePO_4_, under open‐air conditions is proposed. The transition‐metal (Ni) oxidation state in the cathode is reduced by the oxidation of diethylene glycol, facilitating relithiation. Glycol aldehyde formation induces 100% electrode delamination. The electrochemical performance of the regenerated cathodes is recovered, and the regeneration solution is reusable.

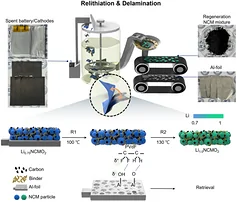

## 1 Introduction

The increasing demand for electric vehicles (EVs) has led to a significant increase in end‐of‐life (EOL) batteries, emphasizing the need for effective recycling processes to address environmental concerns and ensure a sustainable supply of critical materials [1]. Recycling spent batteries reduces environmental waste and contributes to stabilizing the supply–demand balance of essential battery materials, thus promoting a sustainable circular economy [2, 3]. Conventional methods, including pyro‐ and hydrometallurgy, recover Ni, Co, and Li as precursors [4]. Although these methods are industrially feasible, they have disadvantages, including high energy usage, excess greenhouse gas emissions, and significant wastewater generation [5]. These drawbacks highlight the need for alternatives.

To overcome these challenges, direct recycling has emerged as a promising alternative to recover cathode‐active materials without destroying their structure. Compared to conventional methods, direct recycling requires less energy, and lower costs while preserving the electrochemical properties of the battery materials. Owing to these advantages, direct recycling has recently attracted significant attention as a promising strategy for sustainable battery recycling [6]. However, to achieve commercial viability, high‐quality cathode materials must be recovered by minimizing impurities and maintaining cost‐effectiveness [7].

Conventional methods for separating the cathode from Al foil involve typically strong‐base chemical dissolution, crushing, and thermal treatment [8]. However, these approaches have drawbacks, including excessive chemical consumption, Al contamination, and the release of harmful gases. The presence of Al impurities can decrease the energy density and pose risks during cell operation. In particular, an Al content of more than 0.2 wt.% in Ni_0.6_Co_0.2_Mn_0.2_(OH)_2_ can promote the growth of primary particles while inhibiting the growth of secondary particles, ultimately resulting in poor cycling and rate capability [9]. Although previous studies have not addressed Al‐foil contamination, our group evaluated Li_1.0_Ni_0.6_Co_0.2_Mn_0.2_O_2_ (NCM) samples containing fine Al particles, as well as heat‐treated NCM (Figure S1). These samples showed degraded electrochemical performance and poor electrode integrity, particularly after treatment at 700°C (Figure S2). These results highlight the urgent need for a separation method that prevents the formation of Al impurities while preserving the structural integrity of the cathode materials.

A major challenge during long‐term cycling is the degradation of cathode materials, particularly Li deficiencies in the active material (AM), which subsequently leads to reduced electrochemical performance. Li deficiency primarily arises from parasitic reactions, most notably the persistent formation of the solid–electrolyte interphase on the anode, which continuously and irreversibly consumes Li originally stored in the cathode. Consequently, the spent cathodes have significant Li deficiency in their crystal structure, which must be replenished through a regeneration process [10].

Current relithiation methods include solid‐state, molten salt, solvothermal (hydrothermal), and chemical methods. Each method exhibits limitations, such as the requirements for high energy input or an inert atmosphere [10, 11, 12]. Although chemical relithiation, such as deep eutectic solvent (DES) systems, can proceed at room temperature, it often requires an inert atmosphere or highly energy secondary sintering, which limits its practicality. Therefore, future research should focus on chemical relithiation methods that enable rapid Li replenishment under open‐air conditions and mild temperatures to enhance the feasibility of the direct recycling of NCM cathodes.

This study investigated a novel approach for simultaneously achieving electrode delamination and relithiation in an open‐air atmosphere. Diethylene glycol (DEG) was oxidized to glycol aldehyde (GA) during the reaction, enabling the effective separation of Li_x_Ni_0.6_Co_0.2_Mn_0.2_O_2_ (NCM) mixtures (x < 1.0) from Al foil. Simultaneously, Li ions were replenished in the spent NCM structure, reducing the oxidation state of Ni ions. Thus, the regenerated NCM exhibited a structural and compositional state equivalent to that of fresh NCM. Its scalability was demonstrated by the successful regeneration of long‐cycled NCM cathodes retrieved from commercial 2 and 50 Ah pouch cells. This reaction was broadly applicable to mixtures of cathodes with varying Li deficiencies, as well as to other types of cathodes such as Li_x_FePO_4_ (x < 1.0, LFP). Finally, because DEG underwent minimal oxidation owing to the low‐temperature reaction, the polyol solution was reused multiple times simply by adding Li salt.

Our integrated approach simplifies the direct recycling process, promotes environmentally sustainable practices through green chemistry, and enhances feasibility by enabling operation under open‐air conditions with short reaction times. This study offers a practical and sustainable approach for advancing scalable and direct Li‐ion battery (LIB) recycling technologies.

## 2 Results and Discussion

### 2.1 Mechanism of the Integrated Process

**FIGURE 1:**
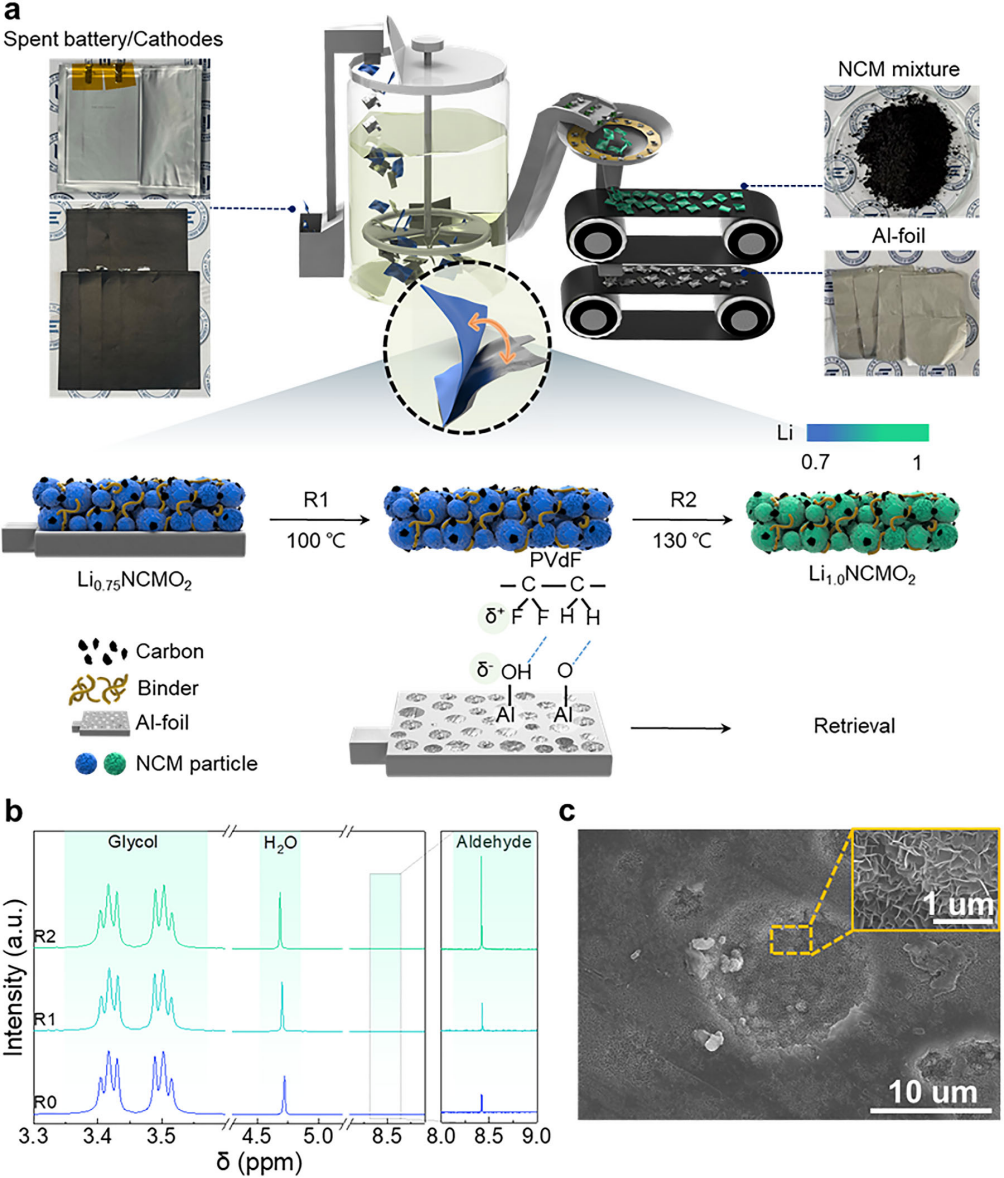
Polyol relithiation and delamination process. (a) Schematic of the process. (b) ^1^H‐NMR spectra of the polyol solution at different reaction temperatures (R0: 60°C, R1: 100°C, and R2: 130°C). (c) FE‐SEM data of the delaminated Al foil (D‐Al), with the inset showing a magnified view of the region indicated by the yellow box.

During the polyol reaction, the electrode underwent simultaneous Li replenishment and Al foil delamination, minimizing impurities and simplifying relithiation (Figure 1a).

(1) $$\begin{array}{cc} & R1:\mathrm{OH}-C_{2}H_{4}-O-C_{2}H_{4}-\mathrm{OH}+O_{2}\\ & \to \mathrm{OH}-CH_{2}-O-CH_{2}-\mathrm{CHO} \end{array}$$

Reaction 1 (R1): the glycol is oxidized to form GA, which disrupts the hydrogen bonds between the Al foil and the polyvinylidene fluoride (PVDF) binder to separate the electrode. The cathode is strongly adhered to the Al foil owing to the hydrogen bonding interactions between the PVDF binder and the surrounding NCM mixture. These interactions are characterized by hydrogen bonding (δH) in Hansen solubility parameters, which reflect the cohesive energy arising from intermolecular hydrogen bonding forces [13]. Thus, GA disrupts the hydrogen bonding between the NCM mixture and Al foil, thereby facilitating interfacial decoupling [14].

(2) $$\begin{array}{cc} & R2:2\mathrm{LiOH}+\mathrm{OH}-C_{2}H_{4}-O-C_{2}H_{4}-\mathrm{OH}\\ & \to \mathrm{LiO}-C_{2}H_{4}-O-C_{2}H_{4}-\mathrm{OLi}+2H_{2}O \end{array}$$

(3) $$\begin{array}{cc} & \mathrm{LiO}-C_{2}H_{4}-O-C_{2}H_{4}-\mathrm{OLi}+O_{2}\\ & \to \mathrm{OHC}-CH_{2}-O-CH_{2}-\mathrm{CHO}+2Li^{+}+4e^{-} \end{array}$$

(4) $$\begin{array}{cc} & Li_{1-x}\left(Ni^{3+/4+}Co^{3+}Mn^{4+}\right)O_{2}+Li^{+}+e^{-}\\ & \to Li_{1}\left(Ni^{2+/3+}Co^{3+}Mn^{4+}\right)O_{2} \end{array}$$

Reaction 2 (R2): Lithiation is initiated by the decomposition of the ─OH groups in DEG, leading to the formation of H_2_O. In this process, Li^+^ remains coordinated with DEG, and the subsequent formation of glycol dialdehyde generates Li^+^ and electrons. These species contribute to the reduced Ni oxidation state, thereby facilitating lithiation of the cathode material [15].

Thus, glycol underwent oxidation to form GA and simultaneously facilitated lithiation via a dehydration‐driven reaction. Upon heating, the ^1^H–NMR spectra showed increased signal intensities corresponding to both H_2_O and aldehyde species. These changes in the spectrum were consistent with the presented reaction pathway.

To validate the proposed mechanism, temperature‐dependent changes in the polyol solution were analyzed using ^1^H‐NMR, as shown in Figure 1b. In the solution, characteristic peaks were observed at 3.35–3.55 ppm (glycol), 4.68 ppm (H_2_O), and 8.37 ppm (aldehyde) [16, 17].

A key advantage of this reaction is that it proceeds without causing metal leaching or aluminum corrosion. As shown in Table S1, inductively coupled plasma atomic emission spectroscopy (ICP‐OES) analysis of the polyol solution confirmed no leaching of the Al foil or transition metals of the cathode. The image of delaminated Al foil (D‐Al), separated by the polyol process, shown in Figure S3, displays a clean surface, whereas the FE‐SEM results reveal a petal‐like structure on the crater surface (Figure 1c). This morphological transformation occurred likely due to the formation of aluminum hydroxide, as indicated by XPS (Figure S4). The feasibility of reusing D‐Al was evaluated by comparing its electrochemical performance with that of pristine Al foil (P‐Al). The NCM electrode fabricated using D‐Al exhibited an electrochemical performance similar to that of P‐Al (Figure S5). However, the presence of surface craters and AlF_3_ coatings on D‐Al indicated that additional post‐processing was necessary to ensure consistent recyclability.

**FIGURE 2:**
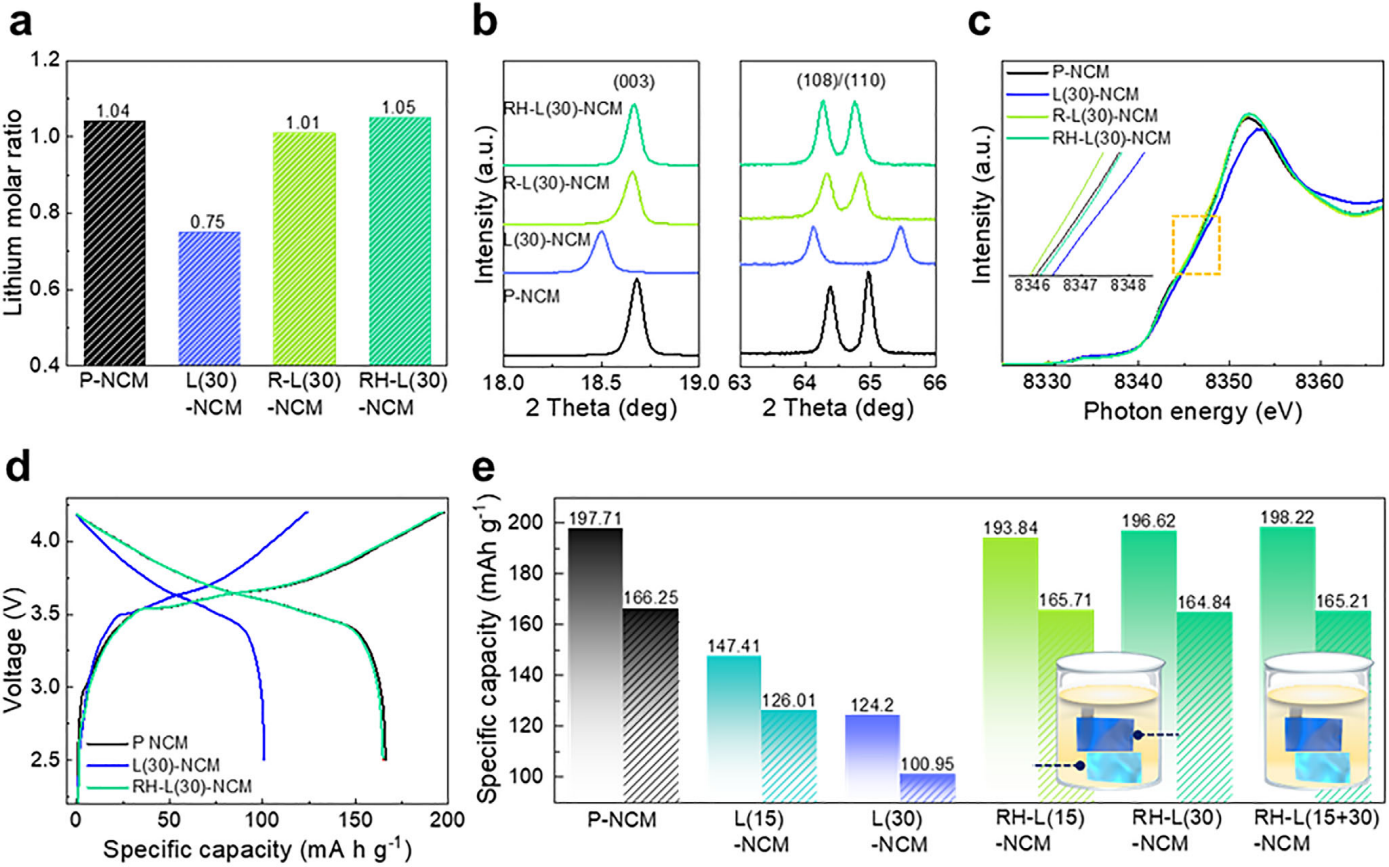
Relithiation characteristics of 30% Li deficiency NCM (L(30)‐NCM) via the polyol reaction. (a) ICP‐OES results for the Li content. (b) XRD results of the (003) and (108)/(110) planes. (c) Ni K‐edge XANES. (d) Full‐cell voltage profile. (e) Uniform Li restoration for various Li deficient electrodes: chart showing the full‐cell charge and specific discharge capacity.

Based on the evaluation of the polyol reaction conditions using the DOD for a 70% cathode (L(30)‐NCM), 130°C for 20 min was identified as the optimal condition for simultaneous restoration and delamination (Figure S6). According to the ICP‐OES results shown in Figure 2a, the Li contents were 0.75 for L(30)‐NCM, 1.01 for the polyol reaction NCM sample (R‐L(30)‐NCM), and 1.05 for the heat‐treated polyol NCM sample (RH‐L(30)‐NCM). The occurrence of higher Li contents in RH‐L(30)‐NCM was caused by the heat treatment following the addition of excess Li salt. The FE‐SEM results confirmed the absence of contamination or morphological changes in the samples (Figure S7). The restoration of Li in the structure was observed by the shift in the (003) plane of XRD pattern (Figure 2b). The lower 2θ shift in L(30)‐NCM was due to Li deficiency, because repulsion between the adjacent oxygen planes increased during delithiation, thereby increasing the *c*‐lattice parameter [18]. Furthermore, the (108)/(110) plane indicated Li deficiency and reflected the lithiation state of the NCM lattice, due to the increased and reduced radii of the transition metal ions of NCM [19]. These results were supported by the lattice and structural parameters obtained from XRD Rietveld refinement (Figure S8 and Table S2). Thus, all samples exhibited a typical *α*‐NaFeO_2_ structure with an R‐3m space group. To accurately determine the Li content and site occupancy, neutron diffraction (ND) was employed to measure the Li content during the polyol reaction (Figure S9 and Table S3). In RH‐L(30)‐NCM, 4.1% of Ni was found in the octahedral sites within the Li layer, along with a slight excess of Li in the transition‐metal layer, a value comparable to the 4.3% observed in P‐NCM. Successful lithiation was confirmed by the nearly identical Li content in the NCM structures: 95.7% in P‐NCM and 95.9% in RH‐L(30)‐ NCM.

To investigate the change in the Ni oxidation state during lithiation, Ni K‐edge X‐ray absorption near‐edge structure spectroscopy (XANES) was performed (Figure 2c). The Ni^3^^+^^/^^4^^+^‐rich oxidation state observed in L(30)‐NCM confirmed that the spectrum of R‐L(30)‐NCM shifted to that of a reduced Ni^2^^+^^/^^3^^+^ state following the polyol reaction. Thus, the polyol reaction altered the Li content by reducing the Ni oxidation state, according to Equation (4). To investigate changes in the Ni oxidation state on the NCM surface during the integrated process, Ni L‐edge photoemission spectroscopy (PES) was conducted (Figure S10). Consistent with the XANES results, a spectral shift toward higher energies in L(30)‐NCM to a spectral shift toward lower energies in R‐L(30)‐NCM was observed. This excessive surface reduction indicates degradation caused by protons from the polyol. However, the alkaline environment provided by the Li salt suppresses H^+^/Li^+^ exchange [20], thereby making relithiation thermodynamically more favorable. Consequently, this minor surface degradation is reversible and is restored to the pristine state using a mild heat treatment at 700 C. Figure 2d confirms the electrochemical performance of RH‐L(30)‐NCM, which exhibits a discharge capacity of 164.8 mAh g^−1^ in the full cell. This result is comparable to that of P‐NCM (166.3 mAh g^−1^). Moreover, RH‐L(30)‐NCM exhibited a capacity retention of 93.7% after 100 cycles, which is similar to that of P‐NCM (93.8%), as shown in Figure S11. Furthermore, the voltage profile obtained under the harsh conditions of an upper cutoff voltage of 4.5 V and at 45°C also demonstrated a performance highly comparable to that of P‐NCM, which offers strong support for these findings (Figure S12). Additionally, the successful restoration of its intrinsic kinetics by the polyol reaction is clearly demonstrated through long‐term dQ/dV analysis (Figure S13) and excellent rate capability up to 10 C (Figure S14).

The varying degradation states of cathodes from various spent batteries resulted in an inhomogeneous Li content, which posed a significant challenge for uniform relithiation. As the polyol reaction is a solution‐based process, it enables homogeneous relithiation [21, 22, 23]. To verify the homogeneous relithiation reaction, samples with 15% and 30% Li deficiency, denoted by L(15)‐NCM and L(30)‐NCM, were uniformly restored using a one‐pot process. As a result, the Li recovered to ∼1.00–1.02, including in the mixed sample (R‐L(15+30)‐NCM, 1.01). The XRD (003) pattern shifted to a higher 2θ, and the full‐cell capacities were 166.3, 165.7, 164.8, and 165.2 mAh g^−^^1^ for P‐, RH‐L(15)‐, RH‐L(30)‐, and RH‐L(15+30)‐NCM, respectively, indicating a 97.7%–98.5% restoration (Figure 2e; Figures S15 and S16).

To determine whether the restored layered structure remained stable or underwent irreversible phase transformations, such as spinel‐ or rock‐salt‐phase formation, more rapidly than the P‐NCM, ex situ SEM and XRD analyses were performed after 100 cycles. As shown in Figure S17, it was difficult to distinguish any distinct differences through the post‐mortem SEM analysis. The post‐mortem XRD patterns (Figure S18) verify that the restored layered structure remained highly stable. The (003) peak positions of RH‐L(30)‐NCM are comparable to that of P‐NCM. Additionally, Spinel‐ or rock‐salt‐type phases were not readily detectable.

In addition to NCM cathodes, LFP cathodes are widely used; however, their low economic value presents significant challenges for recycling. The polyol reaction was applied to evaluate its effectiveness in LFP cathodes. As shown in Figure S19a, the Li content of L(30)‐LFP increased from 0.77 to 1.00 after the polyol reaction, as indicated by the ICP‐OES results.

This recovery was further supported by the XRD patterns shown in Figure S19b, where the FePO_4_ peak—characteristic of Li‐deficient LFP—disappeared in the R‐L(30)‐LFP sample, indicating restoration to a state similar to that of P‐LFP. Evaluation of the electrochemical performance of the cathode using a half‐cell configuration (Figure S19c) revealed an increase in charge capacity, from 106.5 mAh g^−^^1^ for L(30)‐LFP to 152.0 mAh g^−^^1^ for RH‐L(30)‐LFP, which was comparable to the 152.4 mAh g^−^^1^ for P‐LFP. Moreover, RH‐L(30)‐LFP exhibited a capacity retention of 96.5% after 100 cycles, which was similar to that of P‐NCM (96.7%), as shown in Figure S19d. Therefore, as an integrated process, the polyol reaction enables simultaneous delamination and relithiation, making it suitable for direct recycling methods.

### 2.2 Application of Spent NCM Cathodes

To evaluate the applicability of the polyol reaction under practical cathode conditions, two types of spent cathodes were prepared: 2D‐NCM from a 2 Ah cell after 0.5 C 600 charge/discharge cycles and 50D‐NCM from a 50 Ah cell after repeated cycling using the Worldwide Harmonized Light Vehicle Test Procedure (WLTP) Class 3 protocol to simulate severe degradation states.

**FIGURE 3:**
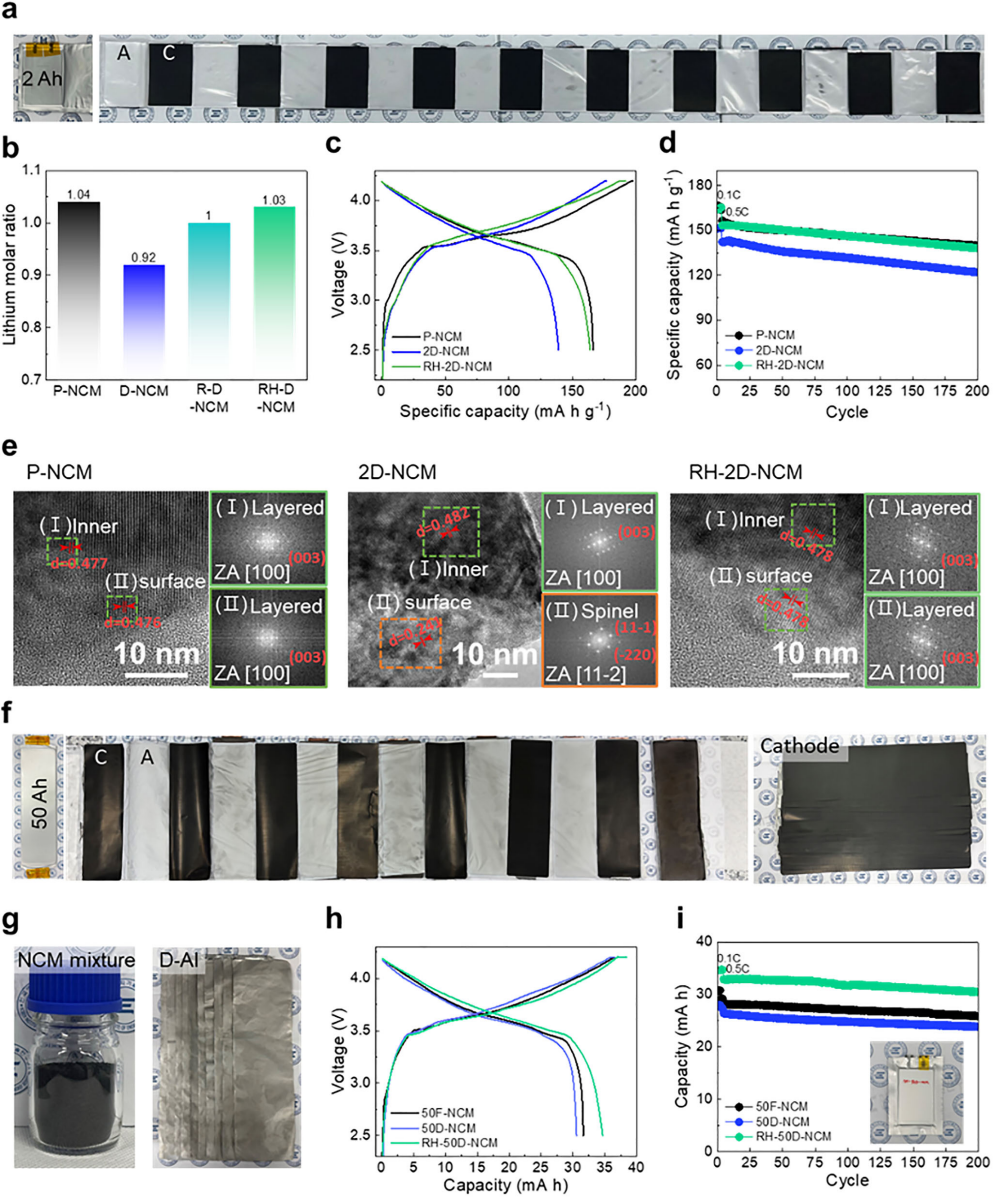
Application of spent NCM cathodes (2 and 50 Ah cells). (a) 2 Ah cell disassembly image (C: cathode, A: anode; in the back of the separator). (b–e) Relithiation results of degraded NCM for a 2 Ah cell (2D‐NCM): b ICP‐OES results, (c) full‐cell electrochemical performance: 0.1 C formation voltage profile, (d) 0.1 C three‐cycle and 0.5 C 200‐cycle cyclability, and (e) FFT patterns obtained from HR‐TEM analysis from left to right: pristine NCM (P‐NCM), degradation of NCM from a 2 Ah cell (2D‐NCM), and relithiation of 2D‐NCM (RH‐2D‐NCM) after heat treatment. (f) 50 Ah cell disassembly image and cathode. (g–i) Relithiation results of degraded NCM for a 50 Ah cell (50D‐NCM): (g) scaled‐up images of the experimental system, (h) voltage profile, and (i) cyclability of the pouch cell.

The 2D‐NCM cathode was retrieved from an 88.3% state of health (SOH), as shown in Figure 3a and Figure S20. Spent batteries typically exhibit severe surface degradation following long‐term cycling driven by mechanisms such as Li deficiency, Ni oxidation, and phase transitions [24], which differ from the degradation observed in electrochemically delithiated cathodes. As shown in Figure 3b–d, 2D‐NCM was successfully restored. The ICP‐OES results confirmed that the Li content of 0.92 in 2D‐NCM was restored to 1.00 in R‐2D‐NCM and further increased to 1.03 for RH‐2D‐NCM after heat treatment. As shown in Figure S21, the FE‐SEM results revealed no significant morphological changes before or after the polyol reaction. The changes in the Ni oxidation state were examined (Figures S22 and S23). Consistent with the L(30)‐NCM results, a spectral shift toward higher energies in 2D‐NCM to a spectral shift toward lower energies in R‐2D‐NCM was observed. Thus, Li‐deficient and long‐cycled NCM, which underwent changes in the Ni oxidation state depending on its Li content, was replenished, even in spent batteries. The structural restoration of Li was confirmed by XRD Rietveld refinement (Figure S24 and Table S4) and ND Rietveld refinement (Figure S25 and Table S5). Both R‐2D‐NCM and RH‐2D‐NCM exhibited a typical *α*‐NaFeO_2_ structure with an R‐3m space group. In RH‐2D‐NCM, 4.2% of Ni was found in the octahedral sites within the Li layer, along with excess Li in the transition‐metal layer, similar to the 4.3% observed in P‐ NCM. The electrochemical performance revealed that in the 0.1 C formation cycle, the initial discharge capacities were 165.1 and 164.1 mAh g^−^^1^ for P‐NCM and RH‐2D‐NCM, respectively. After 200 cycles, the capacity retention of RH‐2D‐NCM was 89.7%, as compared to 89.1% for P‐NCM, thereby demonstrating similar performances. As shown in Figure 3e, High resolution transmission electron microscopy (HR‐TEM) and Fast‐Fourier Transform (FFT) analyses were performed to investigate the surface structural changes after the polyol reaction. The surface of region (II) in P‐NCM underwent structural degradation owing to long‐term cycling, forming a spinel‐like structure similar to that of region (II) in 2D‐NCM. After the polyol reaction, the layered structure was restored, even though RH‐2D‐NCM was subjected to heat treatment (Figure S26).

To further evaluate the recovery performance of batteries at the EV scale, the cathode from a 50 Ah cell (50D‐NCM) degraded by cycling it 32 times using the WLTP model at 25°C to 97.6% SOH was used (Figure 3f, Figure S27). Details of the cathode constituents are presented in Tables S6 and S7 and Figure S28. Furthermore, Figure S29 shows that ICP–OES and electrochemical performance confirm the successful recovery of 50D‐NCM through polyol reaction. In particular, as shown in Figure S29b, despite the initial variation in the Li content across the bimodal cathode, a uniform Li content was achieved after recovery. To evaluate the electrochemical performance, discharge specific capacity was measured using coin full cells, as shown in Figure S29c. The 50D‐NCM exhibited a capacity of 160.5 mAh g^−^^1^, while the RH‐50D‐NCM showed an improved capacity of 181.7 mAh g^−^^1^. These results highlight the effectiveness of the polyol reaction to restore Li uniformly even in commercial cathodes with different Ni contents and particle distributions. As shown in Figure 3g, for scaling up from the laboratory‐scale, 50D‐NCM was mass‐restored using 1 L of polyol solution, yielding 127 g of the NCM mixture. Finally, the restored 50D‐NCM was applied to a pouch cell. This resulted in an improvement in the discharge capacity from 30.6 mAh in 50D‐NCM to 34.6 mAh in RH‐50D‐NCM, as shown in Figure 3h. Moreover, the cells exhibited a capacity retention of 92.3% for the fresh 50 Ah cell cathode in 50F‐NCM and 92.6% in RH‐50D‐NCM after 200 cycles, as shown in Figure 3i. The restoration of NCM in degraded EV cells under low energy and open‐air conditions demonstrates the feasibility of this method for large‐scale applications.

### 2.3 Solution Reusability

**FIGURE 4:**
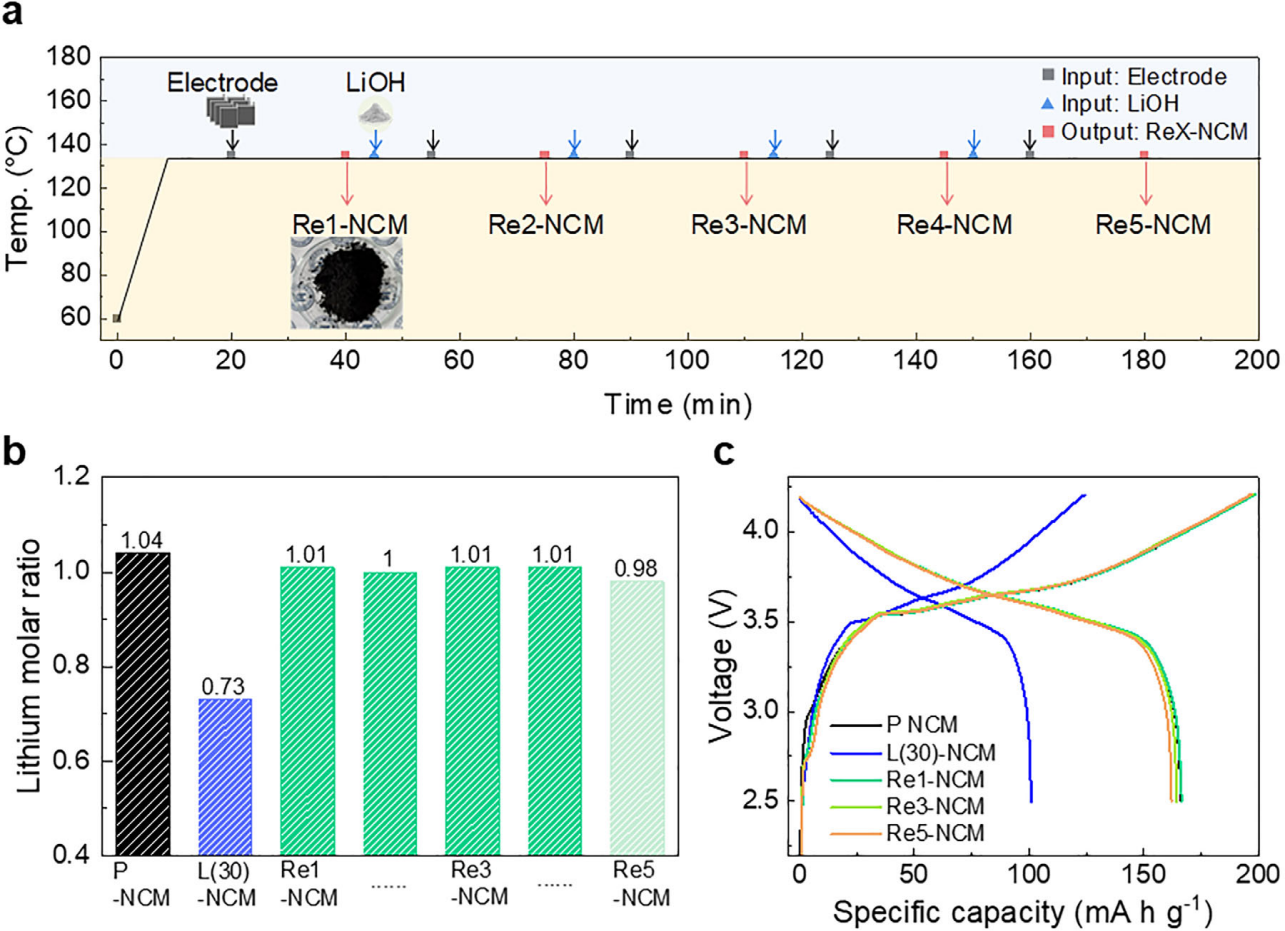
Reuse stability of the polyol solution after reaction. (a) Time‐dependence chart. (b) ICP‐OES results from the cathode after continuous reaction. (c) Voltage profiles of NCM cathodes relithiated in Re1, Re3, and Re5.

To maximize the economic impact of the integrated process, which enables simultaneous electrode delamination and Li restoration, the reusability of the polyol solution was investigated. As shown in Figure 4a, the timeline indicates the time of input of the spent electrodes and Li salt, as well as the recovery point of the replenished NCM. In the NCM cathode of the 30% Li‐deficient 2 Ah cell, the Li deficiency corresponded to 0.86 mmol. In this experiment, when Re1‐NCM was introduced, approximately 80.5% of the Li in the polyol solution had been consumed. To compensate for this loss, additional Li salt was supplied, enabling the preparation of a polyol solution that could replenish Re2‐NCM. This procedure was employed to evaluate the continuous reuse of the polyol solution, and the variations in both the solution and cathodes were monitored over five repeated cycles.

As shown in Figures S30 and S31, the polyol solution gradually darkened owing to the increased concentration of GA generated by DEG oxidation, and in the 3rd cycle, accelerated oxidation of DEG was confirmed by the pronounced evolution of H_2_O. Nevertheless, as indicated in Table S8, although glycol concentration decreased owing to oxidation, a substantial amount of glycol remained in the solution up to the 3rd cycle. From the 5th cycle, a rapid decomposition of DEG was observed, accompanied by a decrease in the generation of H_2_O and GA.

This trend was further confirmed by ICP‐OES and electrochemical performance analysis. The ICP‐OES analysis of the cathodes confirmed a decrease in the Li content in Re5‐NCM (Figure 4b). Consistent with this observation, electrochemical performance analysis revealed that the discharge specific capacities of Re1‐NCM (166.5 mAh g^−^^1^) and Re3‐NCM (164.4 mAh g^−^^1^) were comparable to that of P‐NCM (166.3 mAh g^−^^1^). The Re5‐NCM exhibited a lower capacity of 162.1 mAh g^−^^1^ (Figure 4c), consistent with the ICP‐OES results. However, it was assumed that optimizing the conditions for the continuous reaction could allow more reaction cycles and achieve higher restored performance.

The polyol solution could be reused multiple times, thereby enhancing the process efficiency and economic viability through reduced process steps and ensuring environmental sustainability through efficient energy utilization.

### 2.4 Economic and Environmental Analysis (Techno‐Economic Analysis)

**FIGURE 5:**
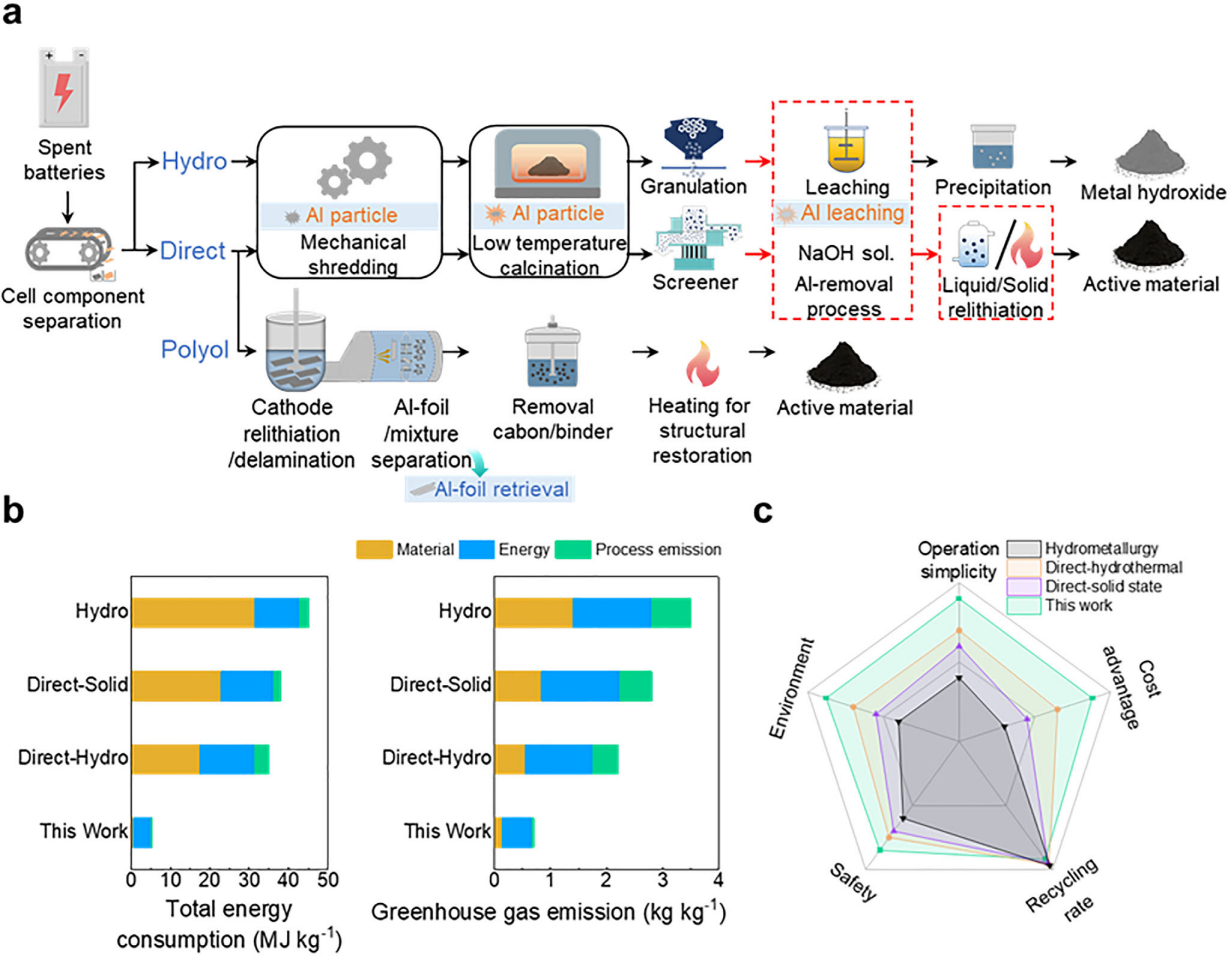
Environmental and economic analysis of different recycling methods. (a) Comparison between the main processes of conventional and direct recycling. (b,c) Economical and environmental analyses: (b) total energy consumption and Greenhouse gas (GHG) emission and (c) radar chart of the main factors affecting the recycling method.

Among the conventional strategies, hydrometallurgy is the most widely adopted method in current industrial practices owing to its ability to efficiently recover critical metals. However, as shown in Figure 5a, this process involves multiple high‐energy steps including leaching, extraction, and precipitation, which result in high energy consumption and significant greenhouse gas emissions. Moreover, conventional direct recycling methods, such as solid‐state and hydrothermal relithiation, present challenges in separating the Al foil. The fine Al particles generated during mechanical shredding are difficult to remove and often contaminate the electrodes. In contrast, the integrated process proposed in this study enables simultaneous Li restoration and Al foil retrieval while preventing the formation of fine Al particles. This integrated low‐temperature process reduces the number of downstream treatment steps and minimizes waste generation. Thus, our proposed efficient recycling process has significant economic and environmental advantages.

As shown in Figure 5b and Tables S9–S15, the total energy consumption of this process is only 5.4 MJ kg^−1^, compared to the 45.3, 38.0, and 12.5 MJ kg^−1^ for hydrometallurgy, direct‐solid, and direct‐hydrothermal methods, respectively. The low material input (1.2 MJ kg^−1^) and minimal energy demand (4.3 MJ kg^−1^) contributed to this low value. Similarly, as shown in the life‐cycle assessment (Figure 5b), the greenhouse gas emissions in the restoration process were reduced significantly to 0.70 kg CO_2_‐eq kg^−1^, while hydrometallurgy and direct‐solid methods generated emissions of 3.50 and 2.80 kg CO_2_‐eq, respectively. These improvements stem from lower emissions across material use (0.31 kg) and energy use (0.35 kg), as well as process emissions (0.040 kg). Furthermore, the radar chart shown in Figure 5c indicates that our method surpasses other methods in multiple categories, including cost advantage, operational simplicity, safety, recycling rate, and environmental performance, thereby underscoring its viability for commercial‐scale implementation. Therefore, the polyol method demonstrates superiority across various categories when compared to conventional and existing direct recycling, hydrothermal, and solid‐state heating methods.

## 3 Conclusion

In this study, we propose a novel integrated polyol‐based regeneration strategy that enables the simultaneous relithiation of spent electrodes and clean delamination of Al foil under open‐air conditions with a low energy input. Our method successfully achieved uniform restoration across cathodes with varying Li contents and structural conditions at low temperatures. As confirmed by ICP‐OES, ND, and XRD, the optimal relithiation conditions of 0.1 m LiOH in DEG at 130°C for 20 min resulted in effective relithiation and restoration. The regenerated cathode materials (RH‐L(15)‐NCM, RH‐L(30)‐NCM, RH‐2D‐NCM, RH‐50D‐NCM, and RH‐L(30)‐LFP) exhibited electrochemical performances comparable to those of the pristine materials (P‐NCM and P‐LFP). The reaction mechanism was elucidated using ^1^H‐NMR, soft‐XAS, and XANES. The GA disrupted the hydrogen bonding between PVDF and the Al foil, enabling delamination, and simultaneously promoted the reduction of Ni oxidation states via Li ions and electrodes, facilitating relithiation into the structure. Furthermore, a techno‐environmental analysis using the Everbatt model confirmed that this method consumed less energy and emitted fewer greenhouse gases than conventional hydrometallurgy or solid‐state relithiation processes. Overall, this simplified and versatile process enables effective control of Al impurities and recovery of high‐purity cathode materials. The demonstrated efficacy of this method on both the NCM and LFP systems suggests its potential as a promising platform for next‐generation closed‐loop LIB recycling.

## 4 Experimental Section/Methods

### 4.1 Specification of Electrodes and Cells

The 2 Ah NCM pouch cells and 1 Ah LFP pouch cell were purchased from Libest Co., Ltd. The ratio of mixed components of the AM, conductive material (CM; carbon black), and binder material (BM; polyvinylidene fluoride (PVDF)) was 94:3:3 for NCM and 89:3:8 for LFP. The mixed components were coated onto both sides of the current collector (Al foil) as 15‐µm‐thick layers. The electrolyte was purchased from Enchem Co., Ltd. and had the following composition: 1 M LiPF_6_ dissolved in a solvent of ethylene carbonate, ethylmethyl carbonate, and diethyl carbonate mixed in a volume ratio of 3:4:3, to which 2 wt.% vinylene carbonate was added. The loading level and density of the single‐sided mixture were 15.1 mg cm^−2^ and 3.0 g cm^−3^ for NCM, respectively, and 8.9 mg cm^−2^ and 1.8 g cm^−3^ for LFP, respectively. In addition, 50 Ah pouch cells were purchased as packs from an automotive parts supplier.

### 4.2 Preparation of Li‐Deficient Cathode and Degraded Cathode

The Li‐deficient NCM cathodes from the 2 Ah cell were cycled at 0.1 C (1 C = 2.175 Ah) in the voltage range 2.5–4.2 V for three cycles. After the 4th discharge cycle, the measured depths of discharge (DOD) were 70% and 85%, and the samples were labeled as L(30)‐NCM and L(15)‐NCM, respectively. The degraded NCM cathodes from the same cell underwent three additional cycles at 0.1 C, followed by 600 cycles at 0.5 C, until the capacity retention reached 88.3%. This sample was referred to as 2D‐NCM. The Li‐deficient LFP cathodes from the 1 Ah cell were cycled at 0.1 C (1 C = 1.173 Ah) in the voltage range of 2.5–3.95 V for three cycles. After the 4th discharge cycle, the measured DOD was 70%, and the sample was labeled as L(30)‐LFP. The pouch cells were disassembled in an Ar‐filled glove box, washed with dimethyl carbonate (DMC), and dried under vacuum for > 12 h. Finally, the L(15)‐NCM, L(30)‐NCM, and 2D‐NCM cathodes were collected.

The 50 Ah cell was discharged to 2.5 V, disassembled using DMC, and subsequently dried in a vacuum chamber at 25°C inside a dry room. This sample was labeled as 50F‐NCM. The degraded commercial cathode from a 50 Ah cell was cycled multiple times under the Worldwide Harmonized Light Vehicle Test Procedure (WLTP) Class 3 protocol [25]. The WLTP is a key method for analyzing EV batteries. It uses dynamic and aggressive acceleration and deceleration profiles to simulate real‐world driving. The WLTP is divided into three classes, with Class 3 specifically for vehicles having a power‐to‐mass ratio (PMR) of over 34 W kg^−^^1^. This class comprises four distinct phases: low, medium, high, and extra‐high speeds. This broad range of load profiles makes WLTP Class 3 applicable to the majority of EVs currently on the market. The 50 Ah pouch cell was disassembled using the same procedure and was referred to as 50D‐NCM.

### 4.3 Recycling Process

The retrievals of the NCM mixtures and delaminated Al foil in the integrated process were conducted under an open‐air atmosphere.

The polyol solution was prepared by dissolving lithium hydroxide (LiOH; Daejung Chemical Co., 99%) in DEG (Sigma Aldrich, 99%) at concentrations ranging from 0.05 to 0.4 m followed by stirring for 12 h at 60°C. The collected cathodes were immersed in a polyol solution and heated in a silicone oil bath at 100°C–160°C for 10–30 min. After cooling to 25°C, the polyol solution was reused after separating the cathode. The cathode was then washed alternately with ethyl alcohol and deionized water to remove any residual polyol solution, thereby separately retrieving the NCM mixture and D‐Al. The NCM mixtures were dried using a convection oven for 12 h at 50°C. The samples were labeled as R‐L(15)‐NCM, R‐L(30)‐NCM, R‐2D‐NCM, R‐50D‐NCM, and R‐L(30)‐LFP. To remove the CM and BM, the samples were washed with N‐methyl‐2‐pyrrolidone (NMP). Subsequently, the NCM samples were heated under an oxygen atmosphere in a tube furnace at 250°C for 6 h and at 700°C (5°C/min) for 3 h with 5 mol% LiOH. The LFP sample was heated under an Ar atmosphere using identical temperature conditions. Finally, the restored AMs were collected and denoted as RH‐L(15)‐NCM, RH‐L(30)‐NCM, RH‐2D‐NCM, RH‐50D‐NCM, and RH‐L(30)‐LFP.

### 4.4 Electrochemical Characterization

The NCM electrode was prepared by mixing the obtained AM, CM (Super P), and PVDF (Solef 5130) in a weight ratio of 94:3:3 in NMP using a mixer (Thinky ARE310) at a high speed (2000 rpm) for 10 min. The slurry was cast onto Al foil (20 µm) and dried in a convection oven at 60°C, followed by overnight drying under vacuum at 80°C. The loading level and density of the NCM electrode were 5.9 mg cm^−2^ and 2.5 g cm^−3^, respectively. The LFP electrode was prepared in a similar manner but at a weight ratio of 89:3:8. The loading level and density of the LFP electrode were 8.4 mg cm^−2^ and 1.8 g cm^−3^, respectively.

The half‐cell electrochemical performance was measured using 2032 coin‐type cells with Li metal (Honjo Metal Co., Ltd., thickness: 500 µm) as the counter electrode and a 17 µm three‐layer (ceramic‐coated polyethylene) separator (W‐scope Korea SC1133) with 60 µL of electrolyte. The electrolyte used was the same as described above. The half‐cells underwent a formation cycle at 0.1 C, 25°C, and 2.8–4.3 V for three cycles, followed by cycling at 0.5 C, 25°C, and 3.0–4.3 V for 100 cycles. The full‐cell electrochemical performance used graphite (Libest Co., Ltd., 4.03 mg cm^−2^, 1.06 g cm^−3^) as the counter electrode, while all other conditions remained identical. The full cells underwent a formation cycle at 0.1 C, 25°C, and 2.5–4.2 V for three cycles, followed by cycling at 0.5 C, 25°C, and 2.8–4.2 V for 100–200 cycles.

### 4.5 Structural and Physical Characterizations

Inductively coupled plasma–optical emission spectrometry (ICP‐OES, Thermo Fisher Scientific, iCAP 7000): The stoichiometric composition was performed using.

X‐ray diffraction (XRD, Rigaku, SmartLab): The powder XRD pattern of the NCM samples was measured using Ni‐filtered Cu Kα_1_ radiation (λ = 1.54059 Å) at 40 kV and 44 mA, with a 2θ scanning range of 10° to 90° in 0.01° steps.

Neutron diffraction (ND): The ND data of NCM samples were obtained using a high‐resolution powder diffractometer at the HANARO facility of the Korea Atomic Energy Research Institute (KAERI). The prepared sample was loaded into an airtight vanadium can in a helium gas atmosphere, and the measurement was conducted in the 2*θ* range of 0–160° with a step size of 0.05 using a constant wavelength of 1.834528 Å.

Rietveld refinement (XRD, ND): The XRD and ND implementations of the FullProf program were used to determine the phase fractions and atomic information from the XRD and ND patterns [26].

X‐ray Photoelectron Spectroscopy (XPS, Thermo Fisher Scientific, NEXSA in GIST Advanced Institute of Instrumental Analysis) using Al Kα as the X‐ray source. The spectrometer was calibrated with respect to the C 1s peak binding energy of 284.6 eV.

Scanning electron microscopy (SEM, Hitachi, S‐4700): The morphology of the electrode was characterized using SEM.

Ion milling instrument (HITACHI, IM‐4000): The cross‐section image of the NCM cathode was obtained using an ion milling instrument under a 6 kV acceleration voltage and discharge current of 400 µA for 90 min.

Electrochemical analysis: The electrochemical performance was measured using a Maccor multichannel battery cycler (Series 4000) for the 2 Ah NCM pouch cell, WonAtech (2PD‐CCJ16) for the CR2032 coin cell, and PNE solution (PEBC05‐400) for the 50 Ah pouch cell.

Soft X‐ray absorption spectroscopy (Soft‐XAS, PAL, beamlines 4D): The valence states and local structures of Ni, Co, and Mn were explored using XAS at the Pohang Accelerator Laboratory (PAL). The transmission mode operated at an electron energy of 3 GeV and a current of 400 mA was used to obtain data for the Ni L‐edge.

Hard X‐ray absorption fine structure (nano XAFS, beamlines 8C): The valence states and bulk structures of Ni, Co, and Mn were explored using XAFS at the Pohang Accelerator Laboratory (PAL). X‐ray absorption near‐edge structure spectroscopy (XANES) and extended X‐ray absorption fine structure spectroscopy (EXAFS) were used at an electron energy of 3 GeV and a current of 400 mA to obtain data for the Ni K‐edge.

High resolution transmission electron microscopy (HR‐TEM, FEI, Tecnai G2 F30 S‐Twin): The samples were prepared using a focused ion beam, operating at 80–300 keV.

Proton nuclear magnetic resonance (^1^H‐NMR, Bruker, AVANCE III HD 400): The polyol solution was used to study the chemical environment and relative abundance of hydrogen atoms in a molecule.

## Author Contributions

Jh. Song: data curation, conceptualization, investigation, formal analysis, visualization, and writing – original draft. S.H. Song: methodology, formal analysis, and writing – review & editing. Dk. Han: data curation, investigation, and writing – review & editing. Jh Noh: data curation, investigation. Jy Kim: investigation, visualization. M.J. Kim: data curation, investigation, and writing – review & editing. Hy Song: data curation, conceptualization, and investigation. J.S. Park: data curation and writing – review & editing. Jk. Kim: conceptualization and writing – review & editing. Jy. Ma: writing – review & editing. Jj. Song: supervision, funding acquisition, data curation, conceptualization, investigation, formal analysis, visualization, and writing – review & editing. J.J. Woo: supervision, funding acquisition, and writing – review & editing.

## Funding

This work was supported by the Korea Institute of Energy Technology Evaluation and Planning (KETEP) and the Ministry of Trade, Industry & Energy (MOTIE) of the Republic of Korea (Nos. RS‐2022‐KP002721 and RS‐2024‐00422151), and the Korea Institute of Energy Research (KIER‐C6‐2405‐20).

## Conflicts of Interest

The authors declare no conflicts of interest.

## Supporting information




**Supporting File**: advs75879‐sup‐0001‐SuppMat.docx.

## Data Availability

The data that support the findings of this study are available on request from the corresponding author. The data are not publicly available due to privacy or ethical restrictions.
