# Supplementary material for: Efficient Direct Recycling of Spent Batteries: Integrated Lithiation and Delamination
Source: Adv Sci (Weinh). 2026 Jun 1:e75879. Online ahead of print. doi: 10.1002/advs.75879 (PMC13335919; doi:10.1002/advs.75879)

Efficient direct recycling of spent batteries: Integrated lithiation and delamination

*Jeonghwan Song^a, b,^*^†^*, Seok Hyun Song^a,^*^†^*, Dokyeong Han^a,c^, Jaehyun Noh^a,c^, Jayoung Kim^a,d^, Min Jeong Kim^a,c^, Hayong Song^a^, Jeong-Sun Park^a^, Jaekook Kim^b^, Jiyoung Ma^a^, Jinju Song^a,*^ and Jung-Je Woo^a,*^*

^a^ Gwangju Clean Energy Research Center, Korea Institute of Energy Research (KIER), Gwangju, South Korea.

^b^ School of Materials Science and Engineering, Chonnam National University (CNU), Gwangju, South Korea.

^c^ Graduate School of Energy Convergence, Gwangju Institute of Science and Technology (GIST), Gwangju, South Korea.

^d^ Department of Materials Science and Engineering, Gwangju Institute of Science and Technology (GIST), South Korea.

^†^ These authors contributed equally to this work.

*Corresponding author Tel: +82-62-717-2402; fax: +82-62-717-2452.

E-mail: Jinju Song: [jinju@kier.re.kr](mailto:jinju@kier.re.kr); Jung-Je Woo: [wooj@kier.re.kr](mailto:wooj@kier.re.kr)

**Contents**

Figure S1 3

Figure S2 4

Figure S3 5

Figure S4 6

Figure S5 8

Figure S6 9

Figure S7 10

Figure S8 11

Figure S9 13

Figure S10 15

Figure S11 16

Figure S12 17

Figure S13 18

Figure S14 19

Figure S15 20

Figure S16 21

Figure S17 22

Figure S18 23

Figure S19 24

Figure S20 25

Figure S21 26

Figure S22 27

Figure S23 28

Figure S24 29

Figure S25 30

Figure S26 33

Figure S27 34

Figure S28 36

Figure S29 38

Figure S30 39

Figure S31 40

[Table S1](#_Toc209625959) 7

[Table S2 1](#_Toc209625960)2

[Table S3](#_Toc209625961) 14

[Table S4](#_Toc209625962) 30

[Table S5](#_Toc209625963) 32

[Table S6](#_Toc209625964) 35

[Table S7 3](#_Toc209625965)7

[Table S8](#_Toc209625966) 41

[Table S9](#_Toc209625967) 42

[Table S10](#_Toc209625968) 43

[Table S11](#_Toc209625969) 44

[Table S12](#_Toc209625970) 45

[Table S13](#_Toc209625971) 46

[Table S14](#_Toc209625972) 47

Table S15 48

**
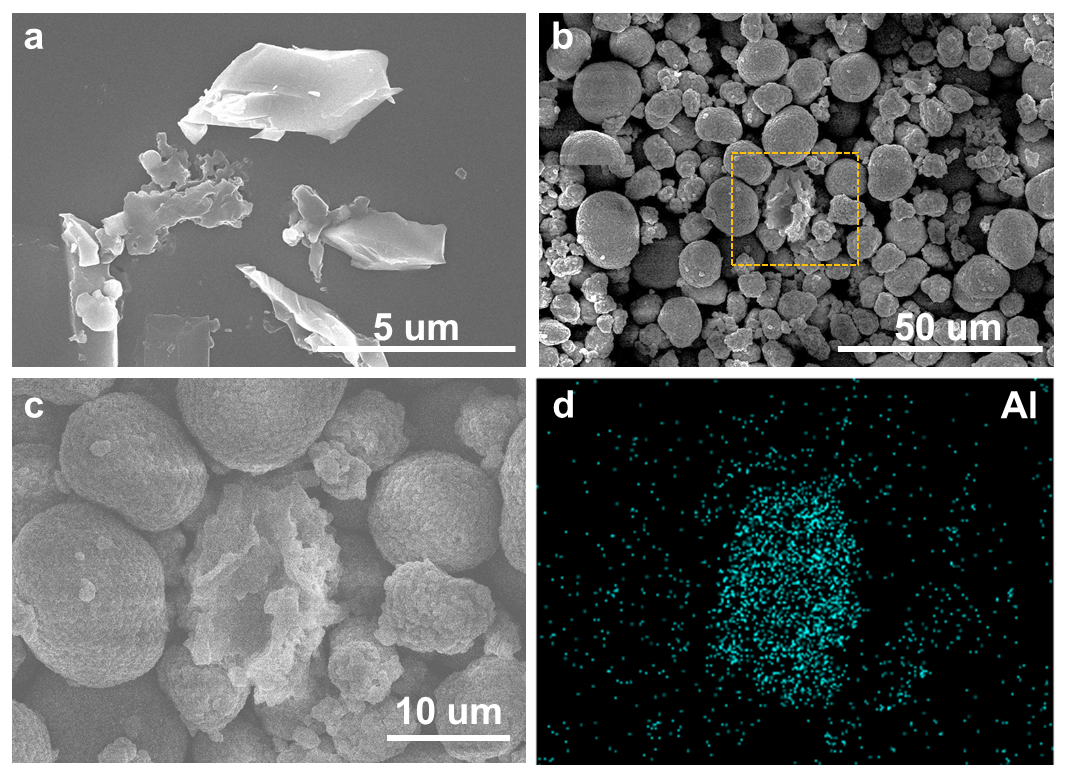
**

Figure S1 FE-SEM data: (a) Al fine particles, (b) blending of Al fine particles and P-NCM after heating at 700 °C, (c) magnified FE-SEM image of the area marked by the yellow box in (b), and (d) EDS mapping the Al signal.

**
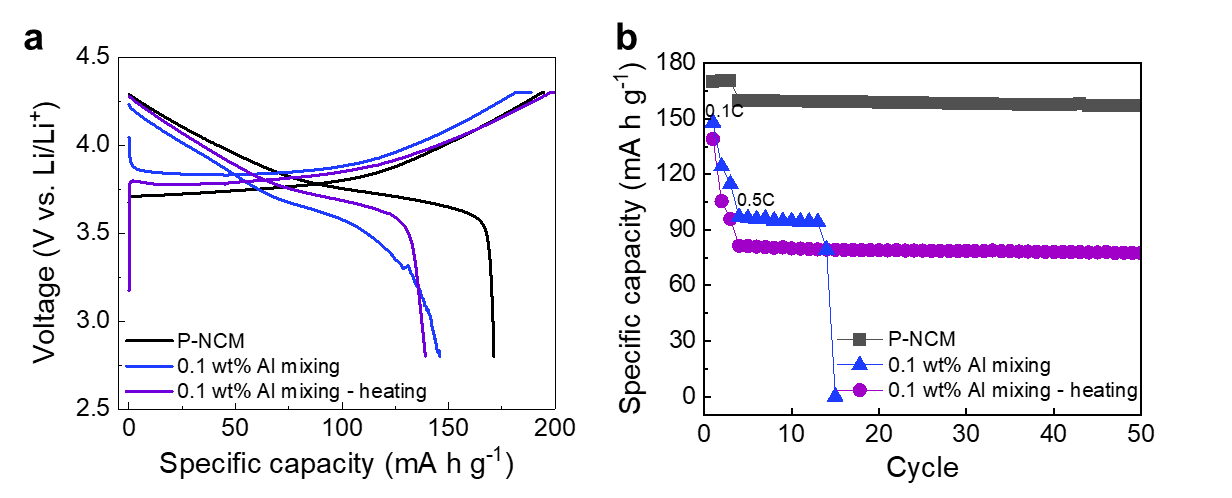
**

Figure S2 Electrochemical performance of P-NCM with Al fine particle contamination; Al fine particles (0.1 wt%) are either physically mixed with P-NCM or mixed by heating at 700 °C. (a) Voltage profile and (b) cyclability.

**
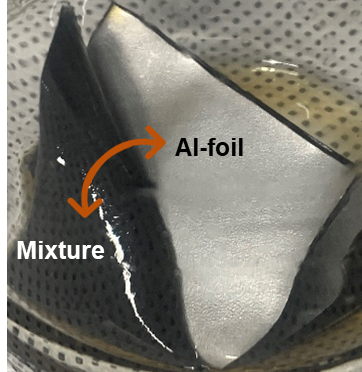
**

Figure S3 Image of Delaminated electrode after polyol reaction: (left) black film: NCM mixture, (right) silver film: D-Al.

**
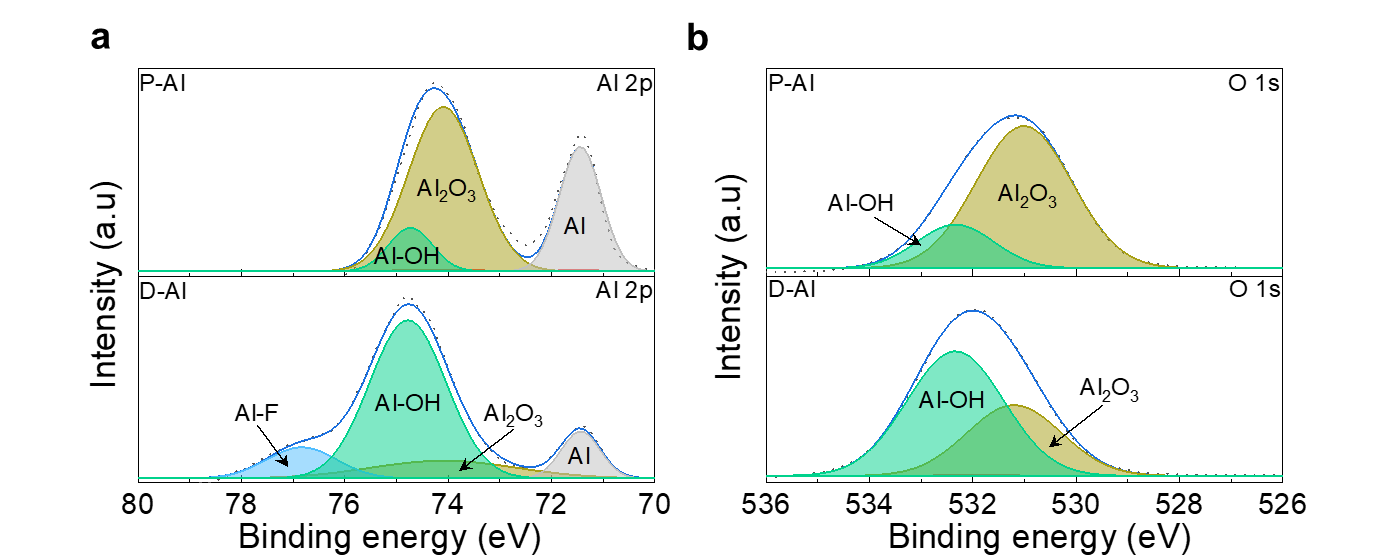
**

Figure S4 XPS spectral data of P-Al and D-Al: (a) Al 2p, (b) O 1s.

Table S1 ICP-OES results analyzing the Li, Ni, Co, Mn, and Al contents in P-NCM, RH-L(30)-NCM and the pre- and post-reaction polyol solutions.


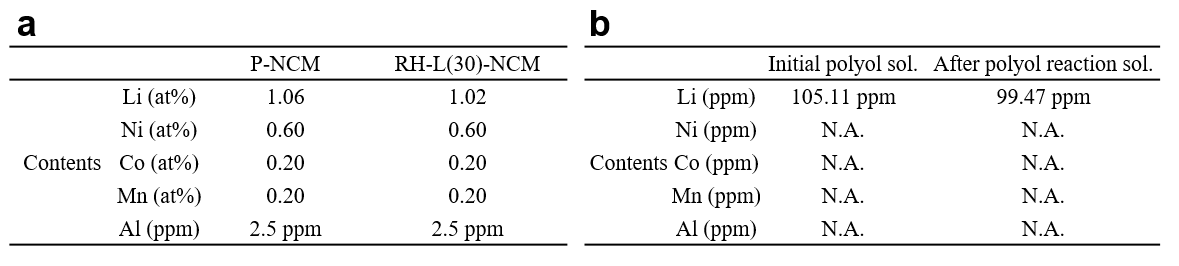


**
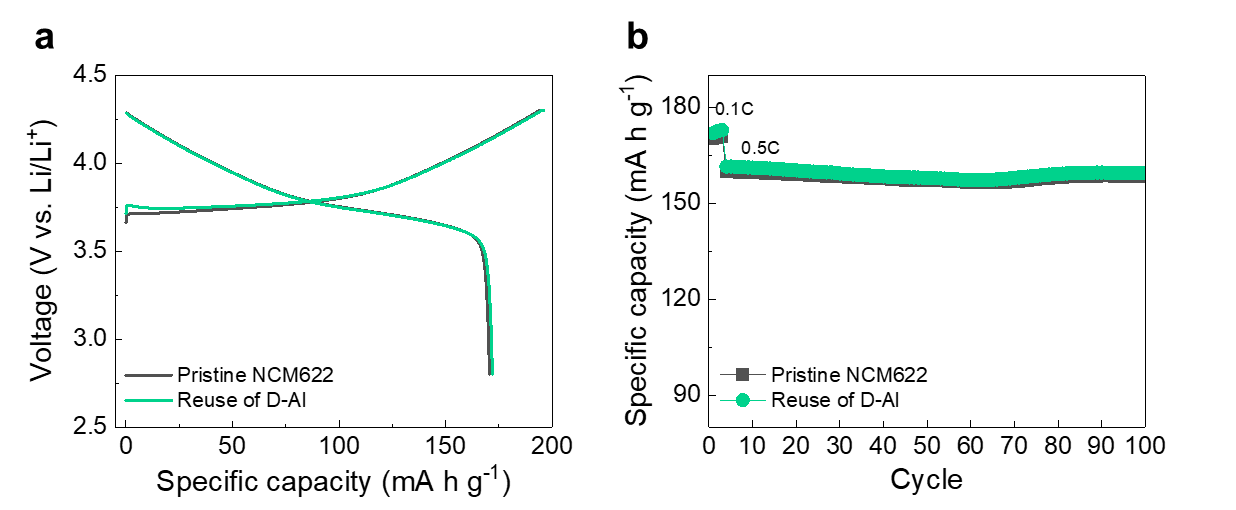
**

Figure S5 Electrochemical performance of reused D-Al: (a) voltage profile, (b) cyclability.

**
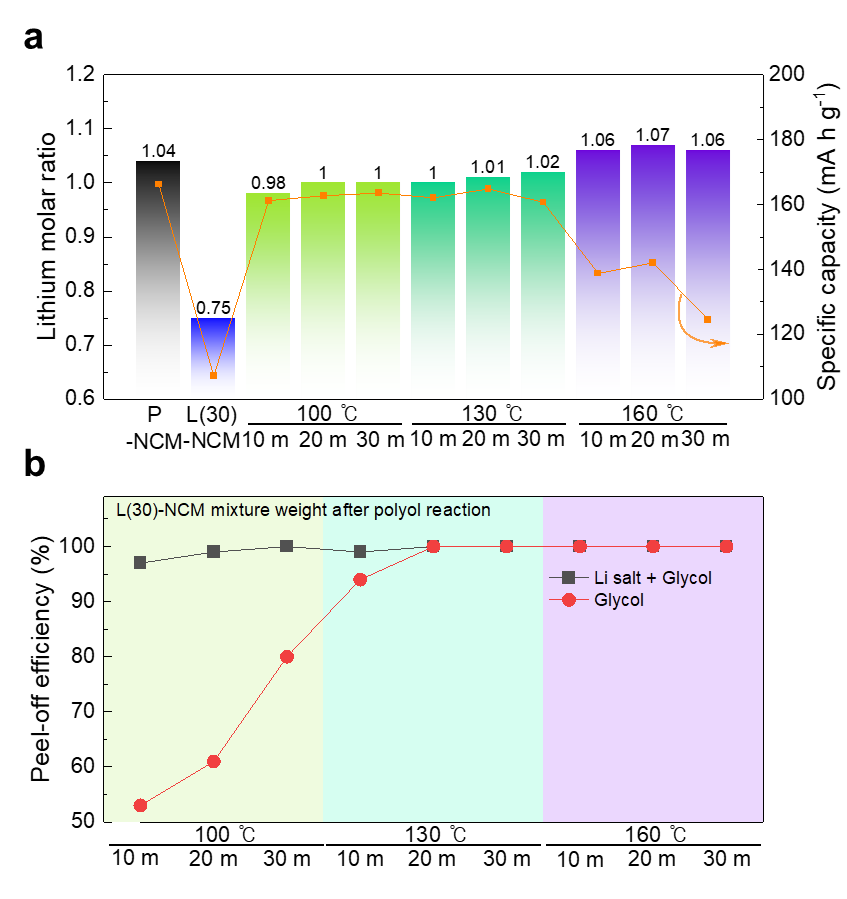
**

Figure S6 Results under different polyol reaction conditions: (a) Comparison of Li content and discharge specific capacity of coin full cells measured by ICP-OES (b) Peel-off efficiency of L(30)-NCM under polyol treatment with and without Li salt.

**
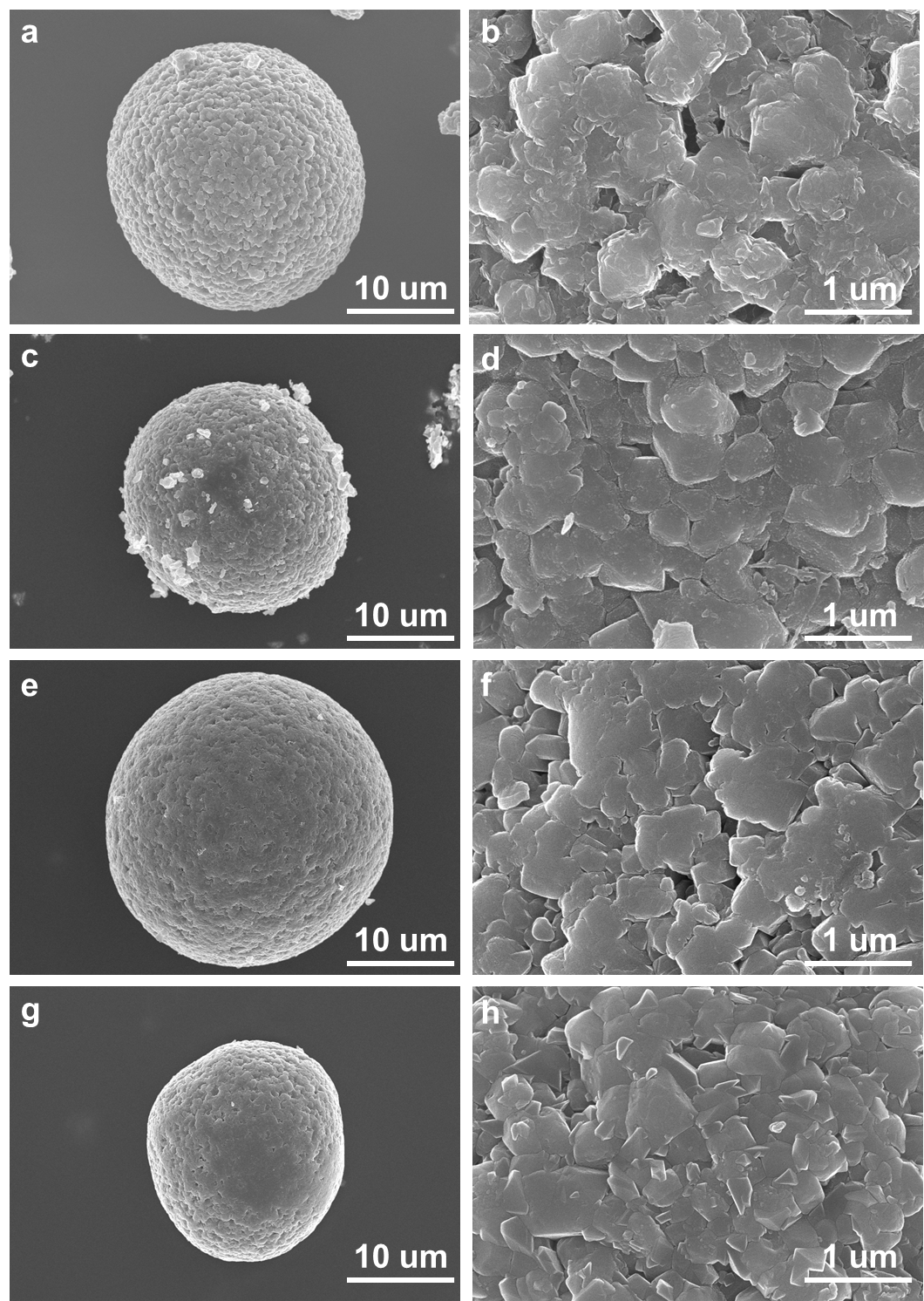
**

Figure S7 FE-SEM images showing the morphology during the polyol treatment of L(30)-NCM: (a, b) P-NCM, (c, d) L(30)-NCM, (e, f) R-L(30)-NCM, (g, h) RH-L(30)-NCM.

**
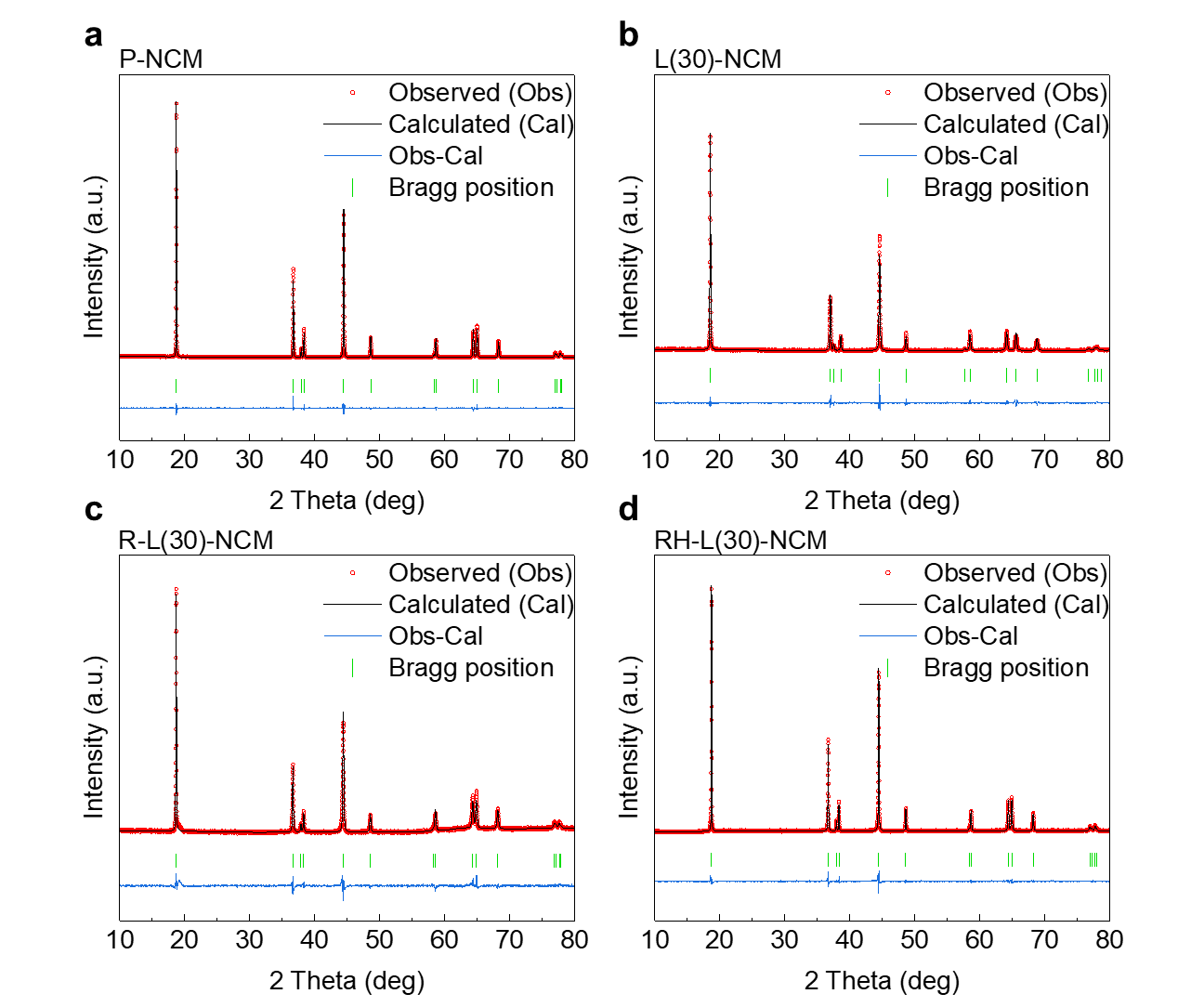
**

Figure S8 Rietveld refinement of X-ray diffraction (XRD) patterns of (a) P-NCM, (b) L(30)-NCM, (c) R-L(30)-NCM, and (d) RH-L(30)-NCM.

Table S2 Detailed structural information from the Rietveld refinement of X-ray diffraction (XRD) patterns.


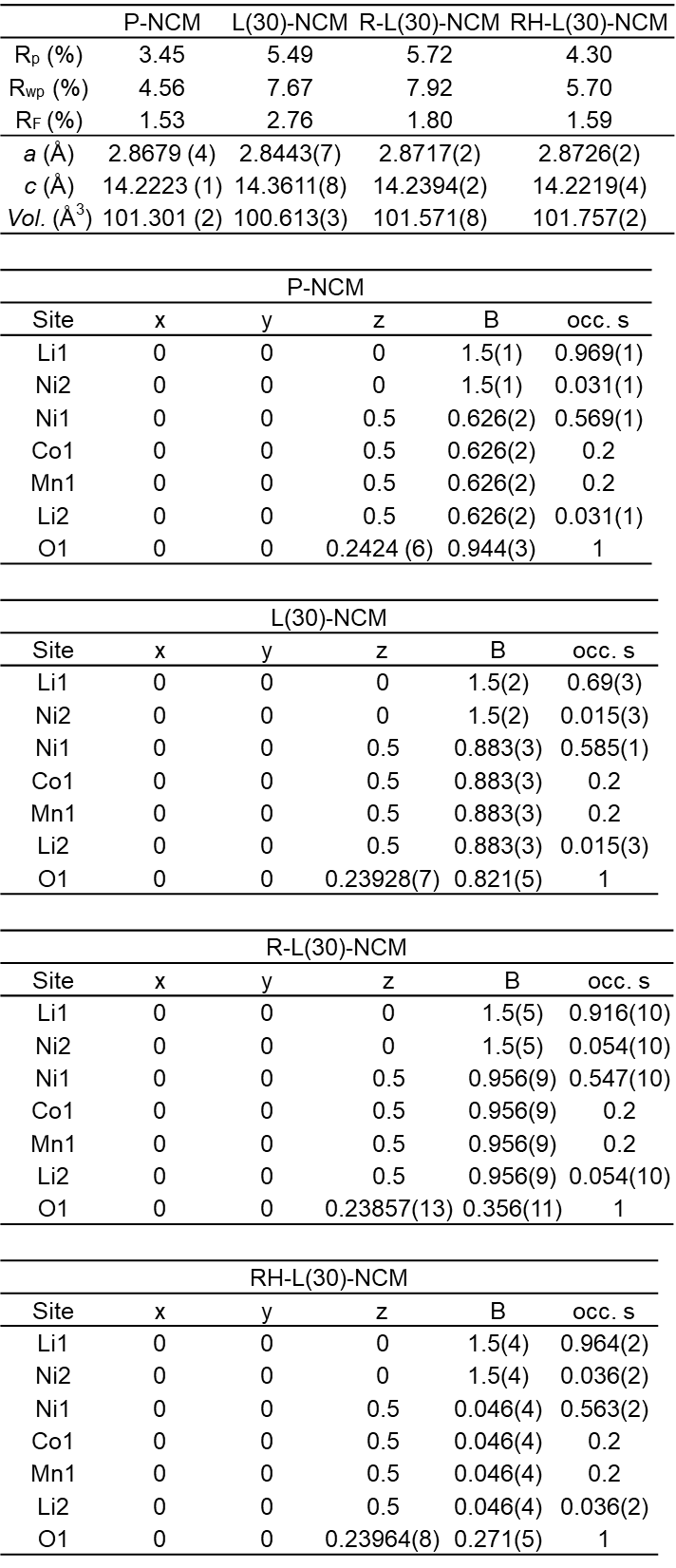


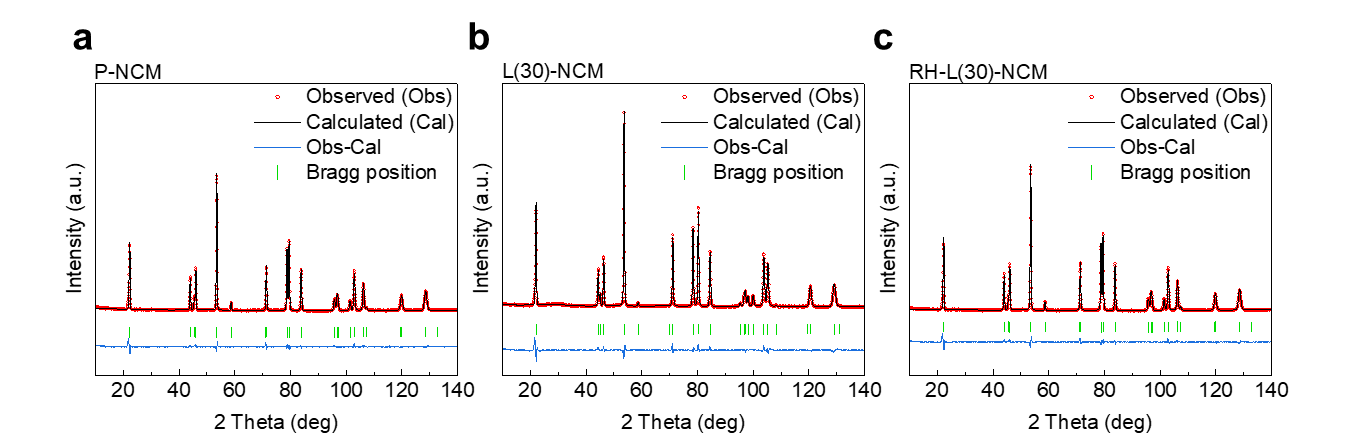


Figure S9 Rietveld refinement of neutron diffraction (ND) patterns of (a) P-NCM, (b) L(30)-NCM, and (c) RH-L(30)-NCM.

Table S3 Detailed structural information from the Rietveld refinement of neutron diffraction (ND) patterns.


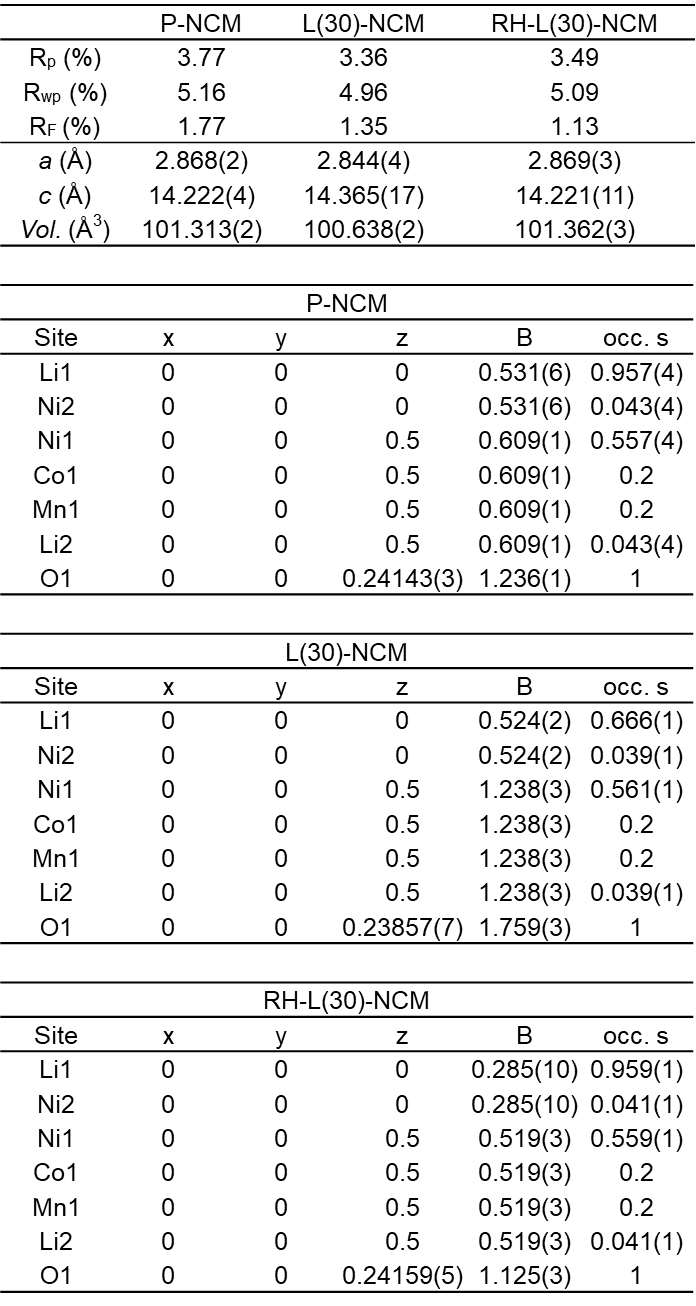


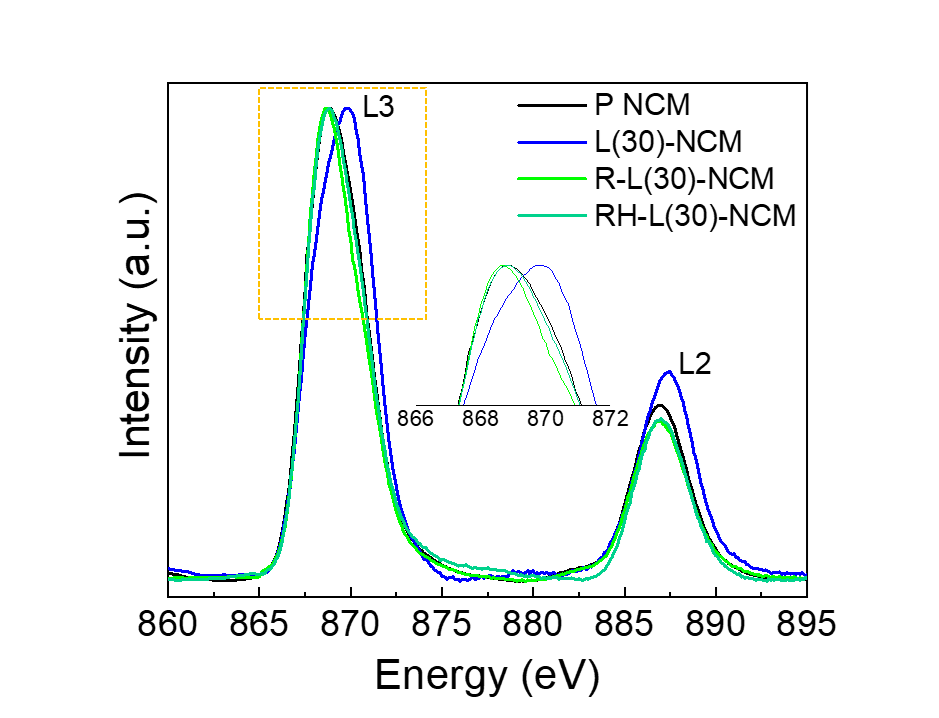


Figure S10 Ni L-edge PES results for the relithiation characteristics of L(30)-NCM.


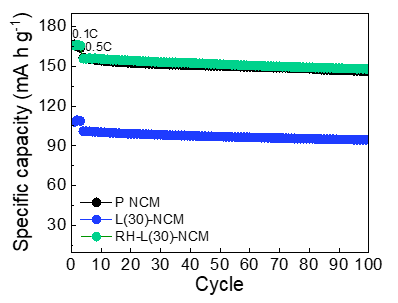


Figure S11 Electrochemical performance: cyclability of RH-L(30)-NCM.

Figure S12 Electrochemical performance evaluated under harsh conditions of the restored RH-L(30)-NCM: upper cut-off voltage of 4.5 V (a) voltage profile and (c) cyclability, and at 45 ℃ atmosphere (b) voltage profile and (d) cyclability.


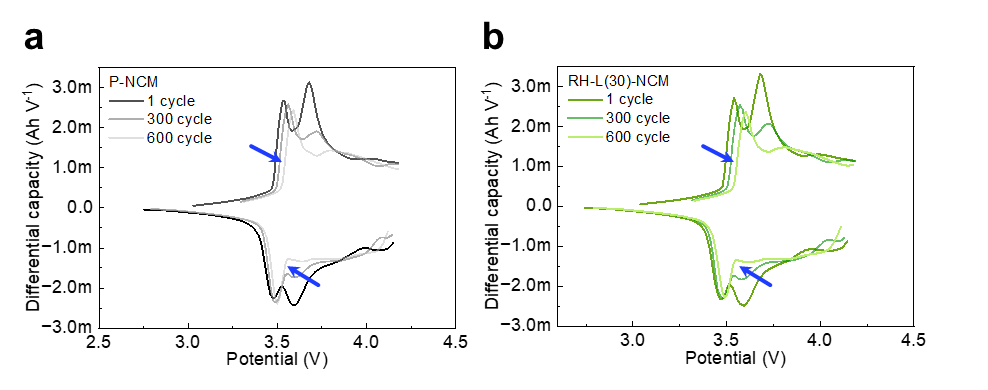


Figure S13 Corresponding differential capacity (dQ/dV) curves for (a) P-NCM and (b) RH-L(30)-NCM at the 1^st^, 300^th^, and 600^th^ cycles at 0.5 C.


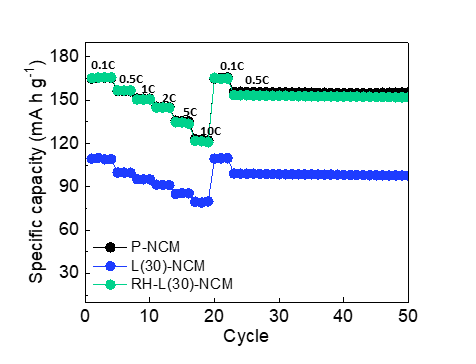


Figure S14 Electrochemical performance: rate capability of RH-L(30)-NCM.


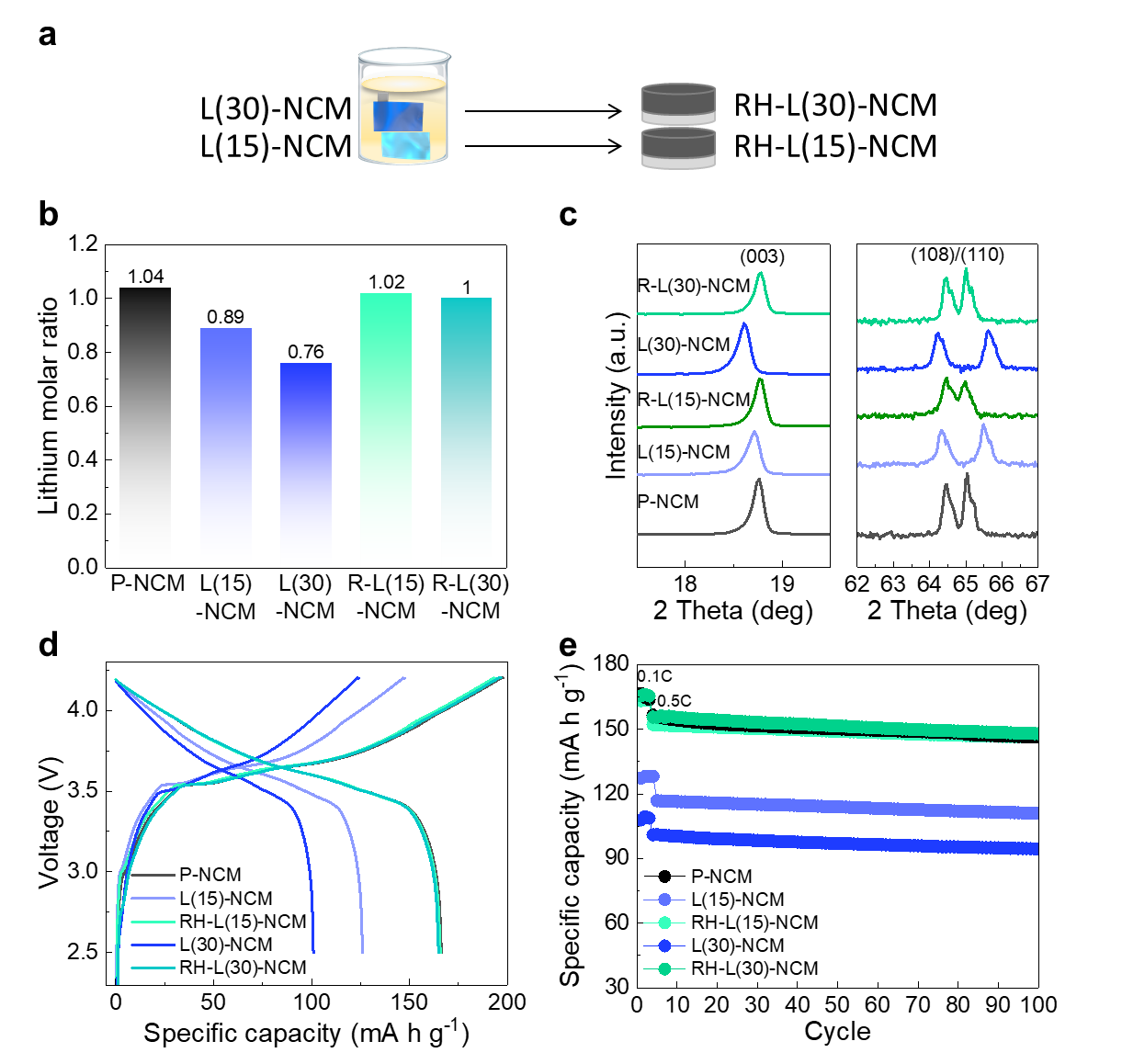


Figure S15 Uniform relithiation results of L(15)-NCM and L(30)-NCM: (a) experimental scheme, (b) ICP-OES results, (c) XRD results of (003) and (108)/(110) planes, (d) voltage profile, and (e) cyclability.


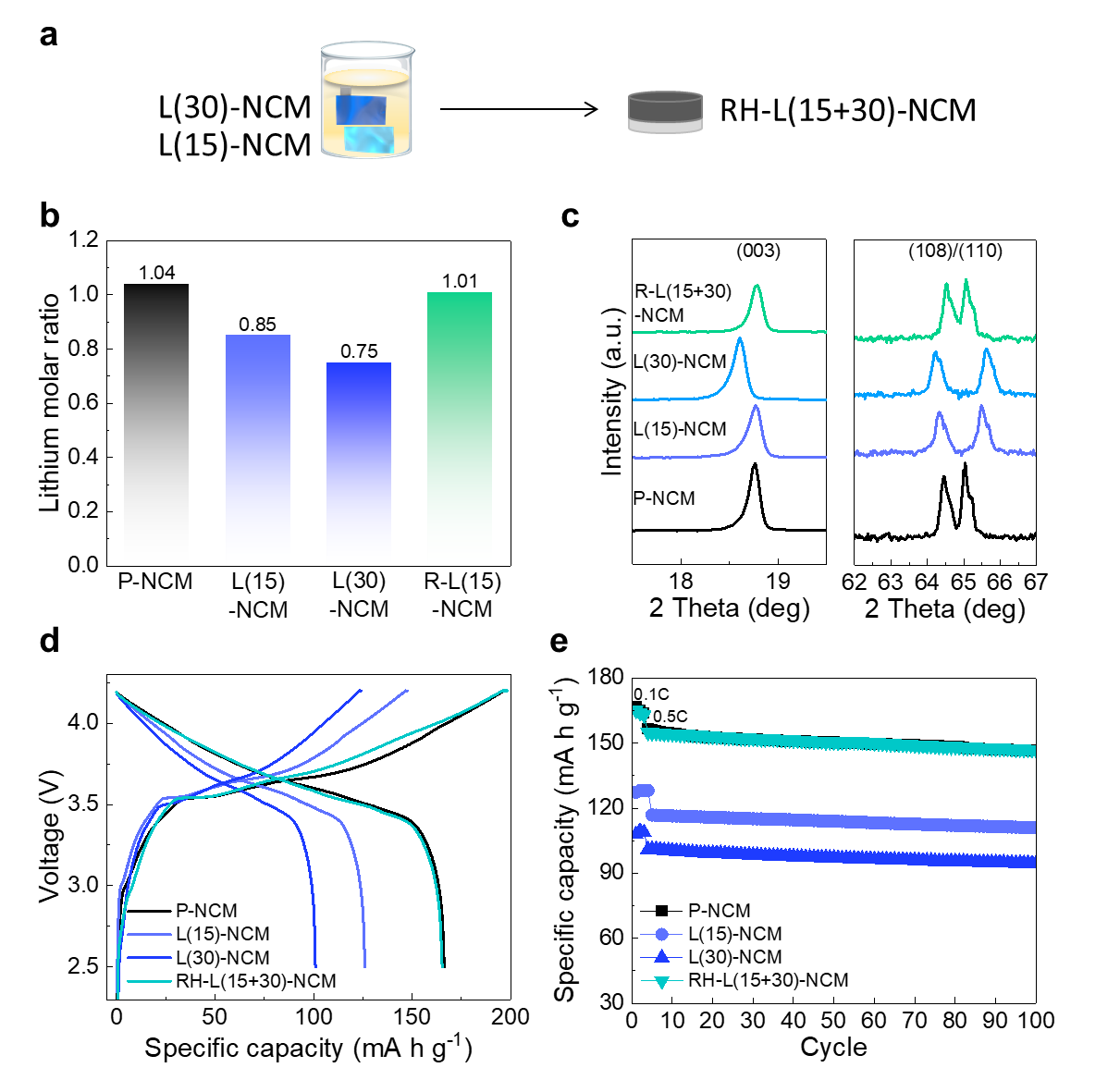


Figure S16 Uniform relithiation result of L(15)-NCM and L(30)-NCM: (a) experimental scheme, (b) ICP-OES results, (c) XRD results of (003) and (108)/(110) planes, (d) voltage profile, and (e) cyclability.


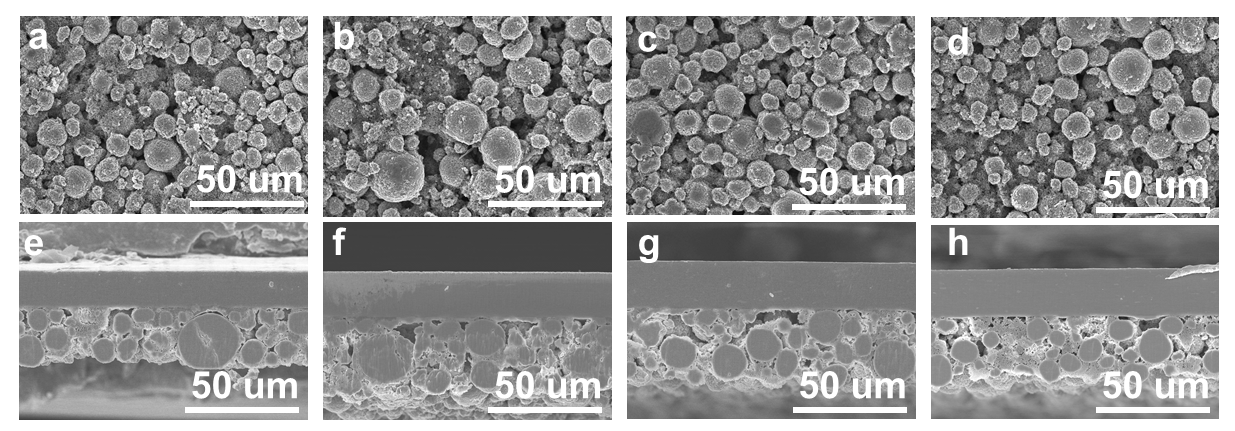


Figure S17 Surface (a–d) and cross-sectional (e–h) SEM images of the cathodes before and after 100 cycles at 1.0 C: (a, e) P-NCM, (b, f) cycled P-NCM, (c, g) RH-L(30)-NCM, and (d, h) cycled RH-L(30)-NCM.


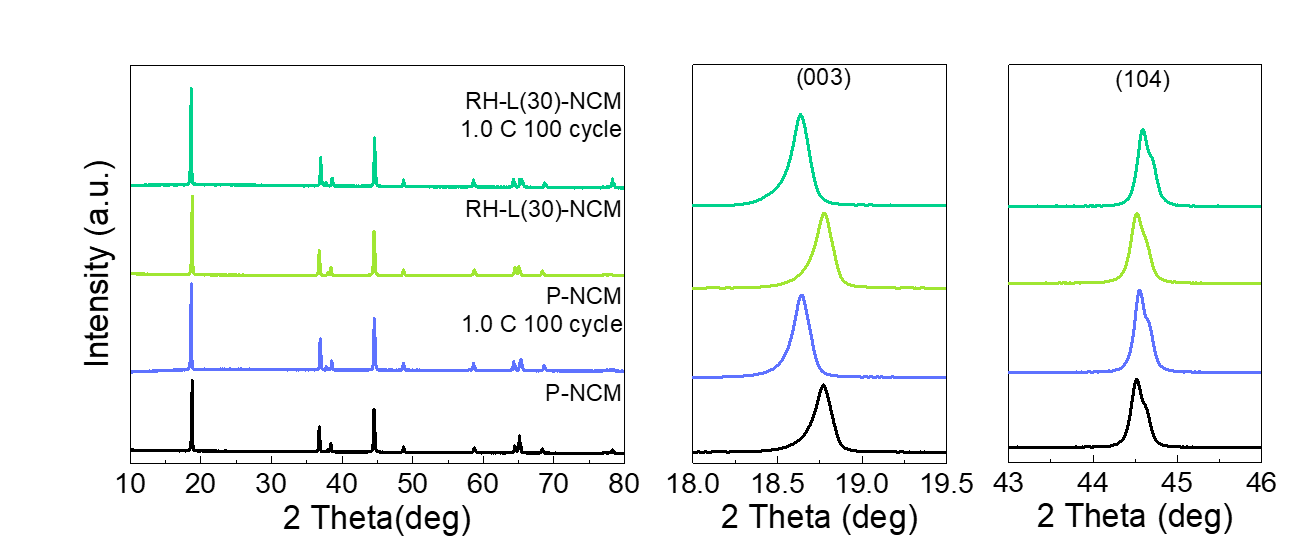


Figure S18 Post-mortem XRD patterns of P-NCM and RH-L(30)-NCM from coin full-cells; (a) after the 1st cycle and (b) after the 100th cycle at 1.0 C.


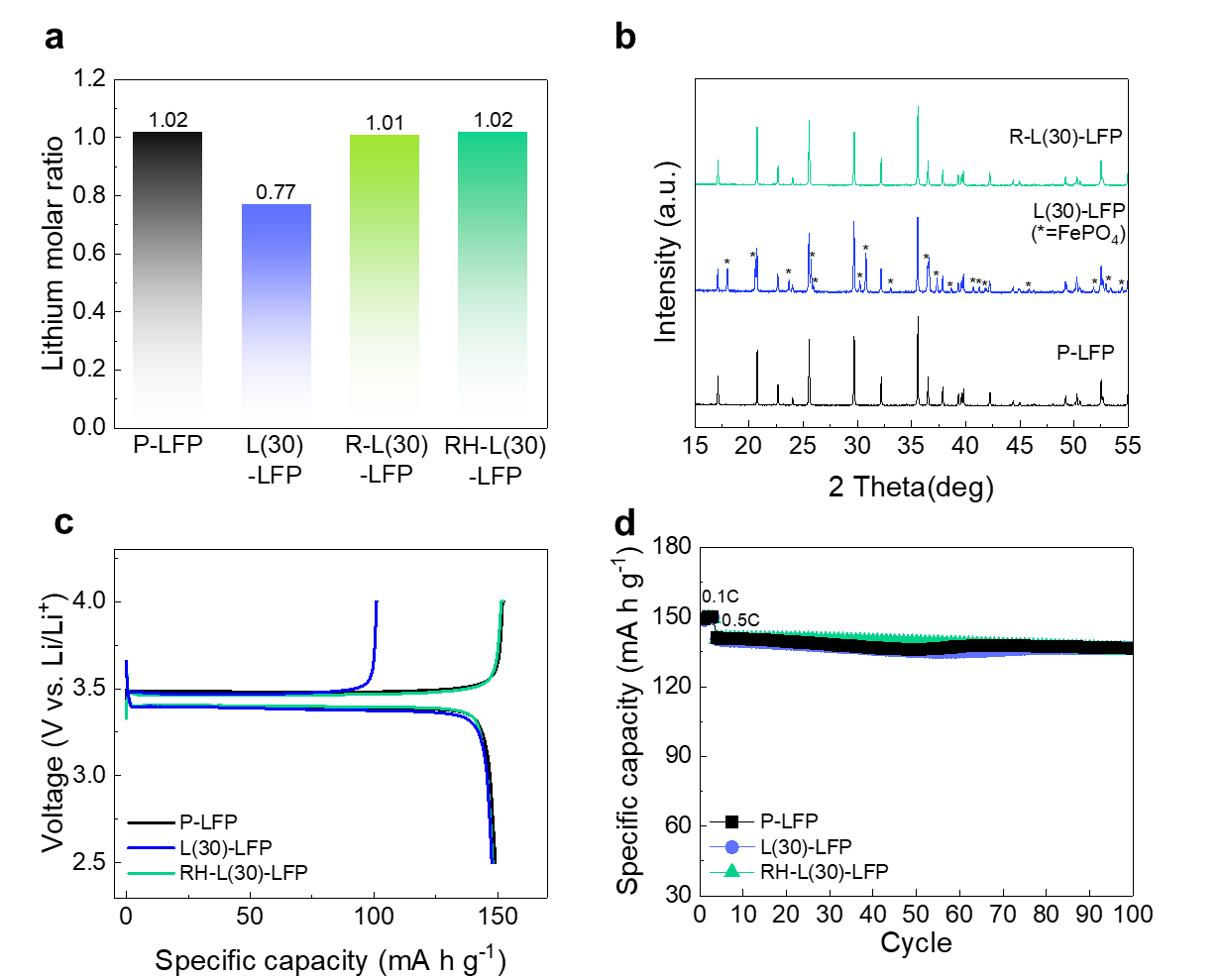


Figure S19 L(30)-LFP relithiation results: (a) ICP-OES results, (b) XRD patterns, (c) voltage profile, and (d) cyclability.

**
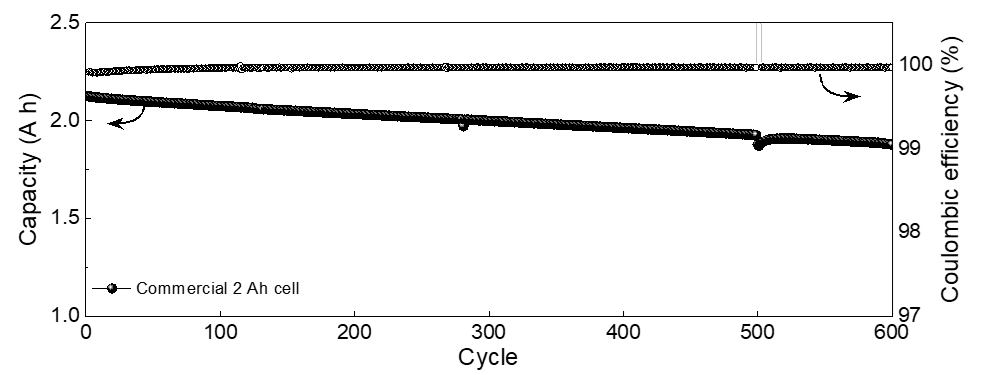
**

Figure S20 Cycling profile of the 2 Ah cell operated for 600 cycles to obtain a degraded cathode, SOH = 88.3%.


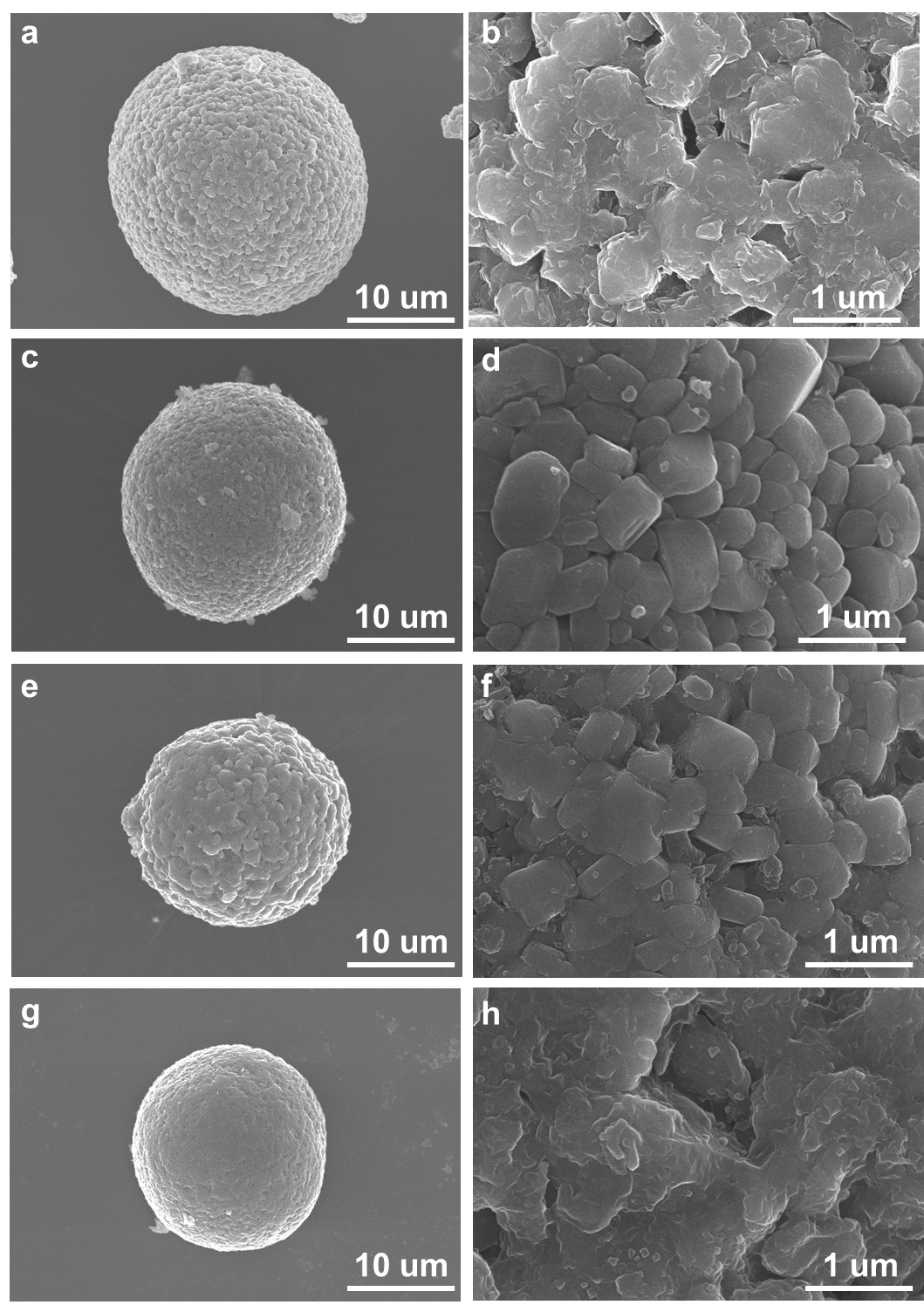


Figure S21 SEM results showing the morphology during the polyol treatment of 2D-NCM: (a, b) P-NCM, (c, d) 2D-NCM, (e, f) R-2D-NCM, (g, h) RH-2D-NCM.


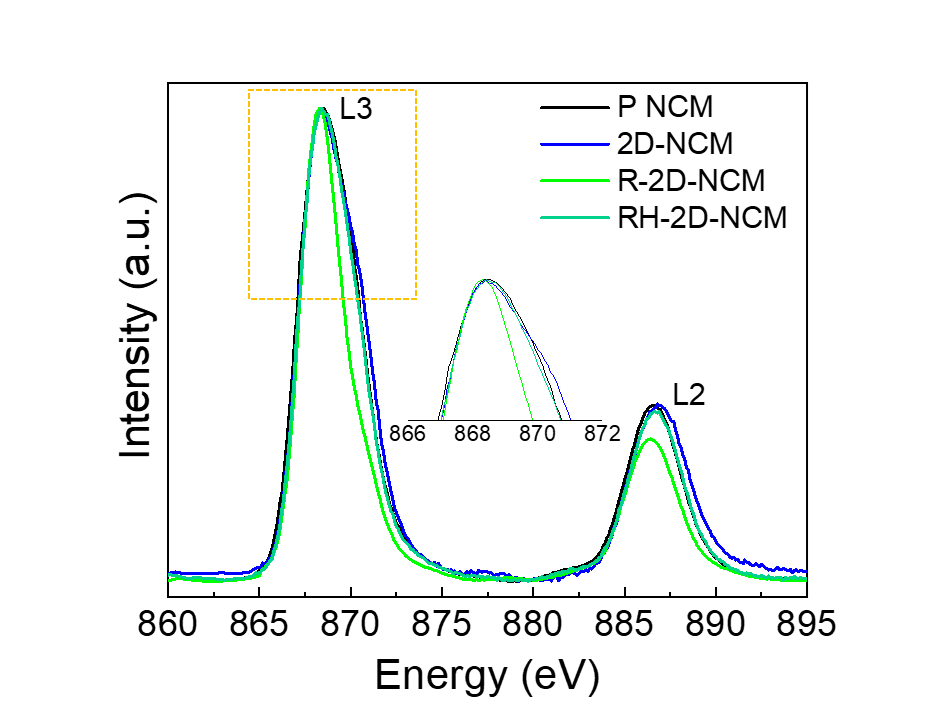


Figure S22 Ni L-edge PES results for the relithiation characteristics of 2D-NCM.

Figure S23 Ni K-edge XANES spectra for the relithiation characteristics of 2D-NCM.


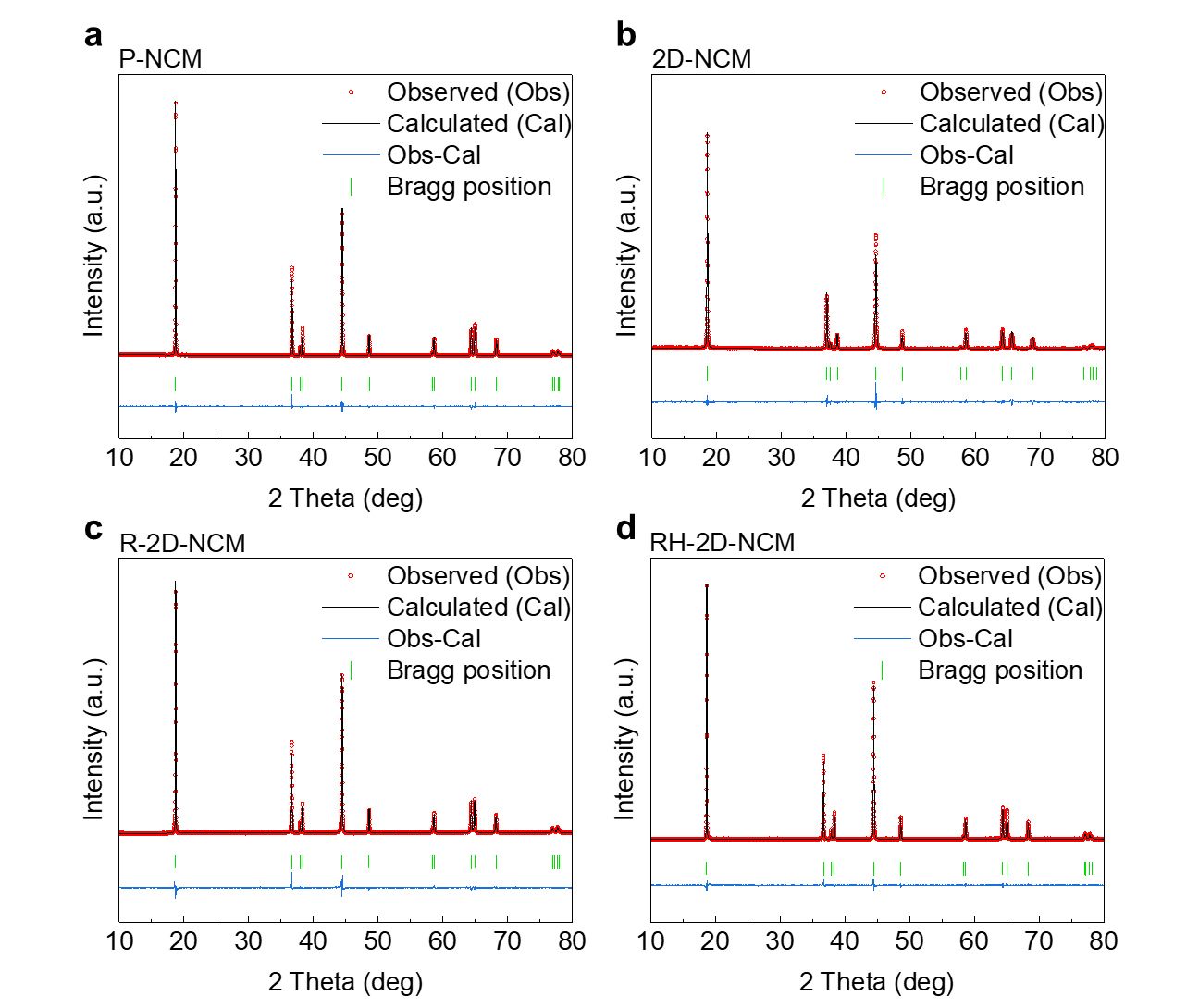


Figure S24 Rietveld refinement of X-ray diffraction (XRD) patterns of (a) P-NCM, (b) 2D-NCM, (c) R-2D-NCM, and (d) RH-2D-NCM.

Table S4 Detailed structural information from the Rietveld refinement of X-ray diffraction (XRD) patterns.


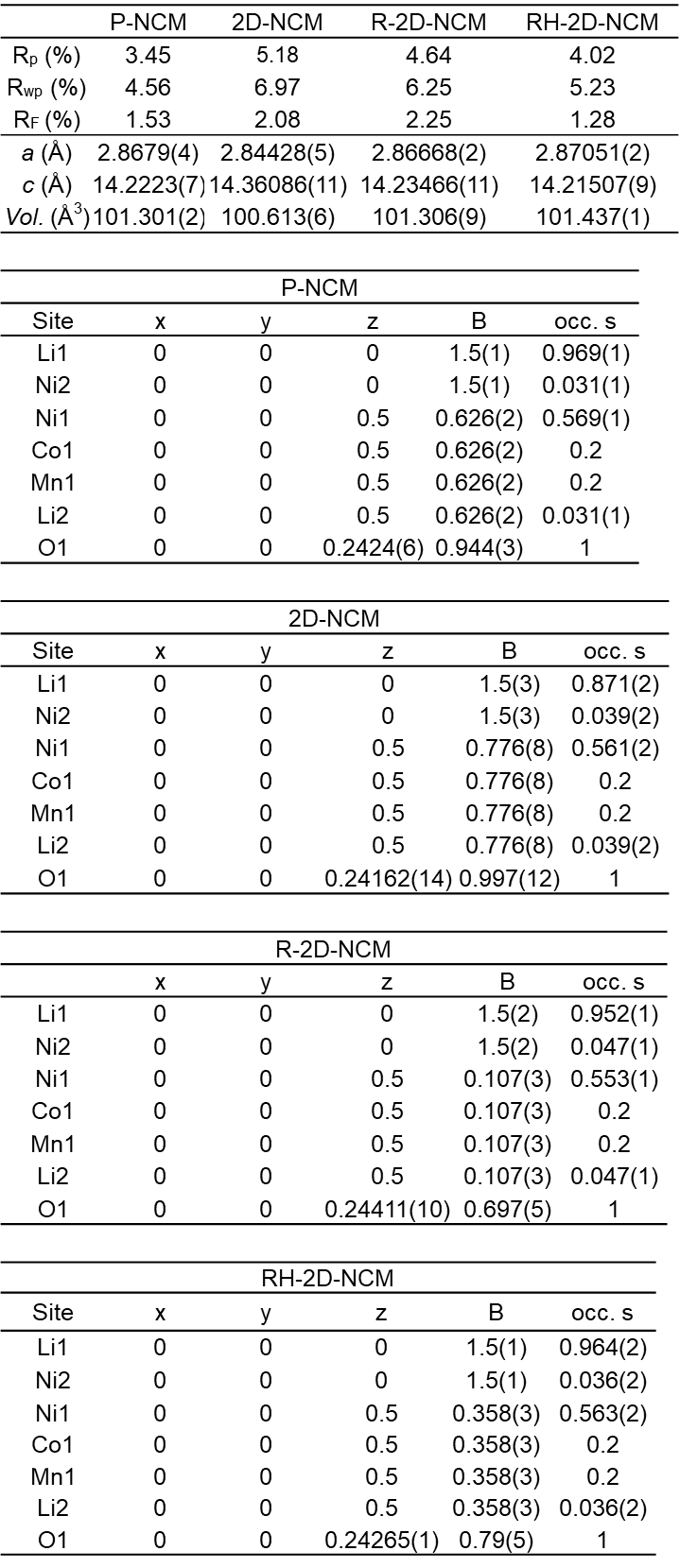


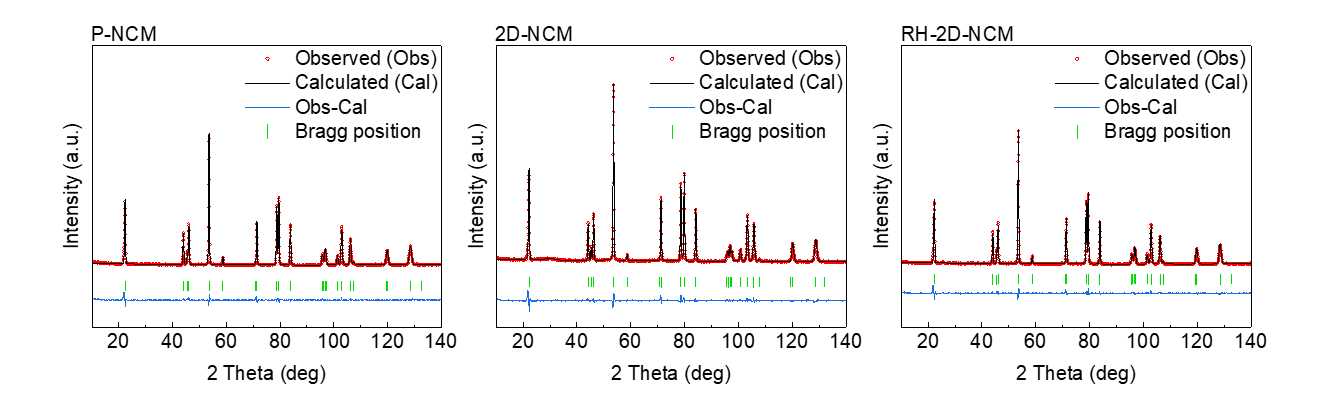


Figure S25 Rietveld refinement of neutron diffraction (ND) patterns of (a) P-NCM, (b) 2D-NCM, and (c) RH-2D-NCM.

Table S5 Detailed structural information from the Rietveld refinement of neutron diffraction (ND) patterns.


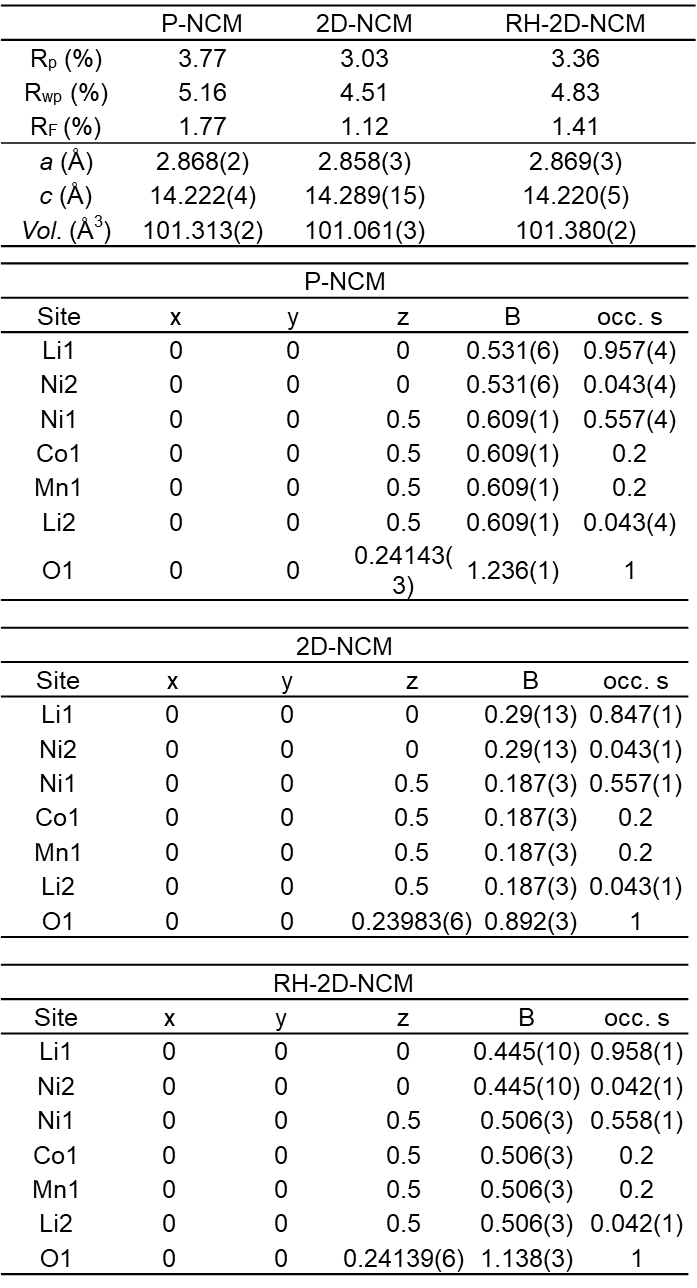


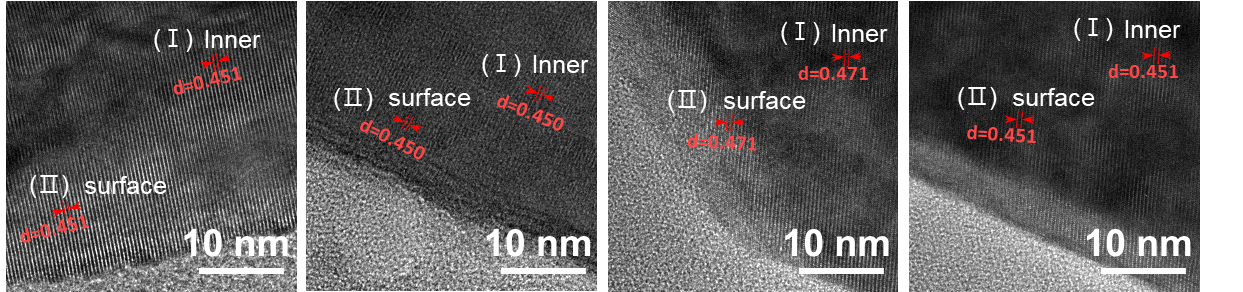


Figure S26 HR-TEM results obtained from various points on RH-2D-NCM.


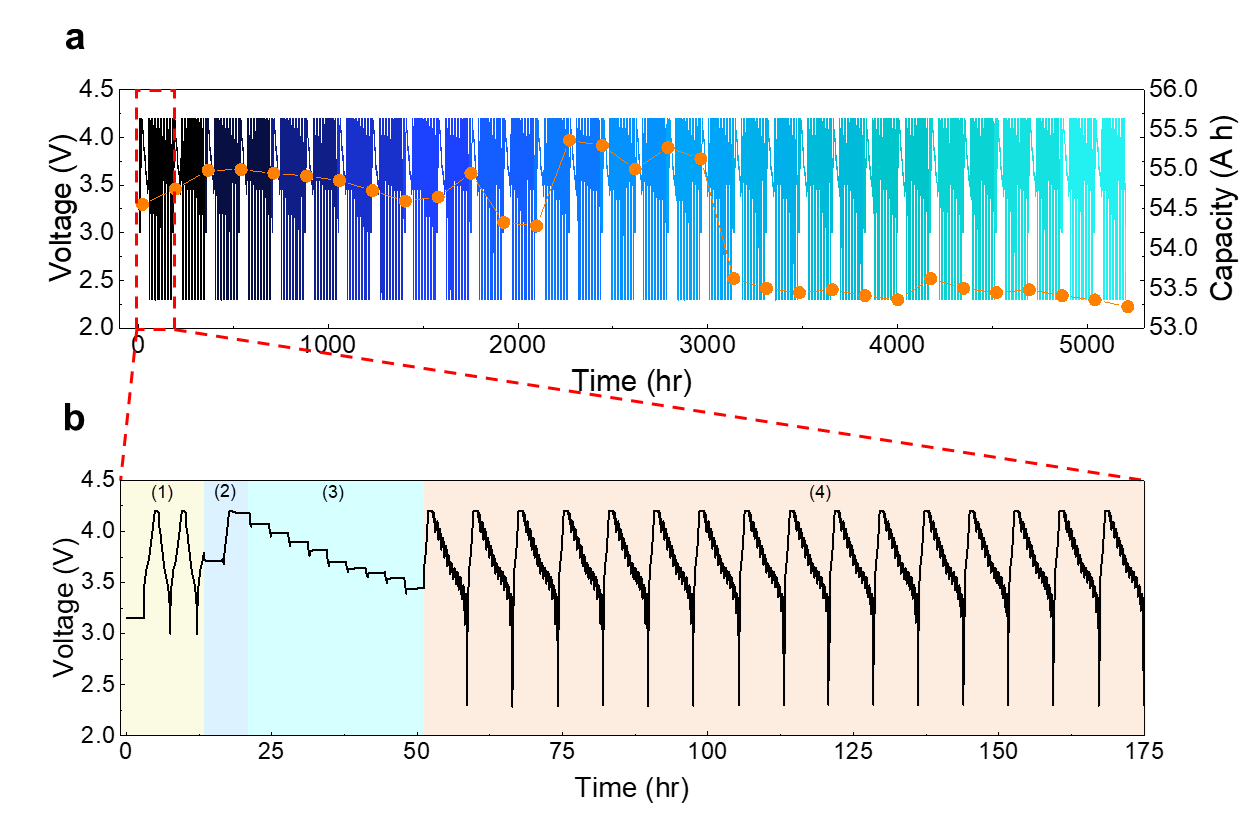


Figure S27 (a) Degradation cycling profile of 50 Ah cell under the worldwide harmonized light vehicle test procedure (WLTP) class 3 protocol, (b) section (1): 0.5 C capacity check, section (2): direct current internal resistance (DC-IR), section (3): repeated pulse test (RPT), section (4): simulation drive.

Table S6 ICP-OES results for 50D-NCM.


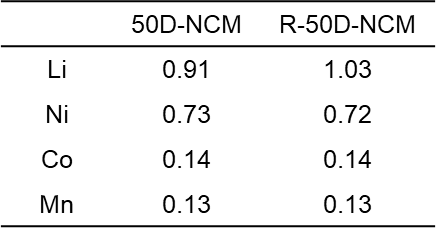


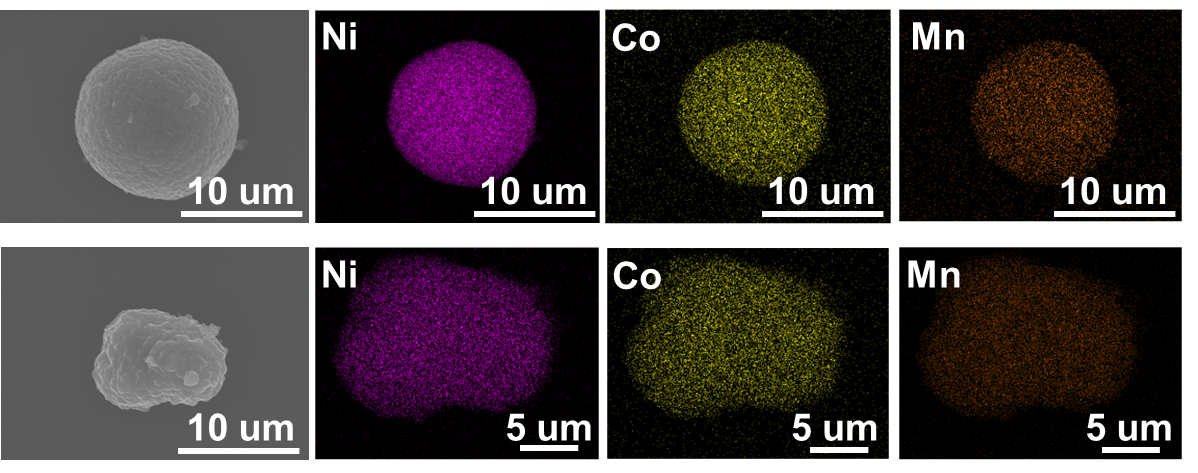


Figure S28 FE-SEM/EDS results for 50D-NCM particles.

Table S7 EDS results for 50D-NCM particles.


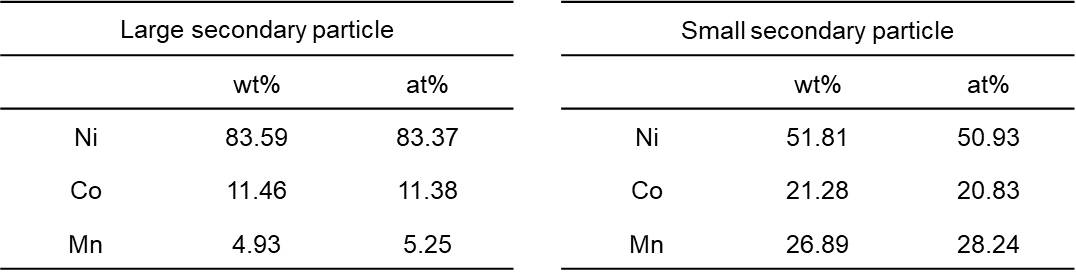


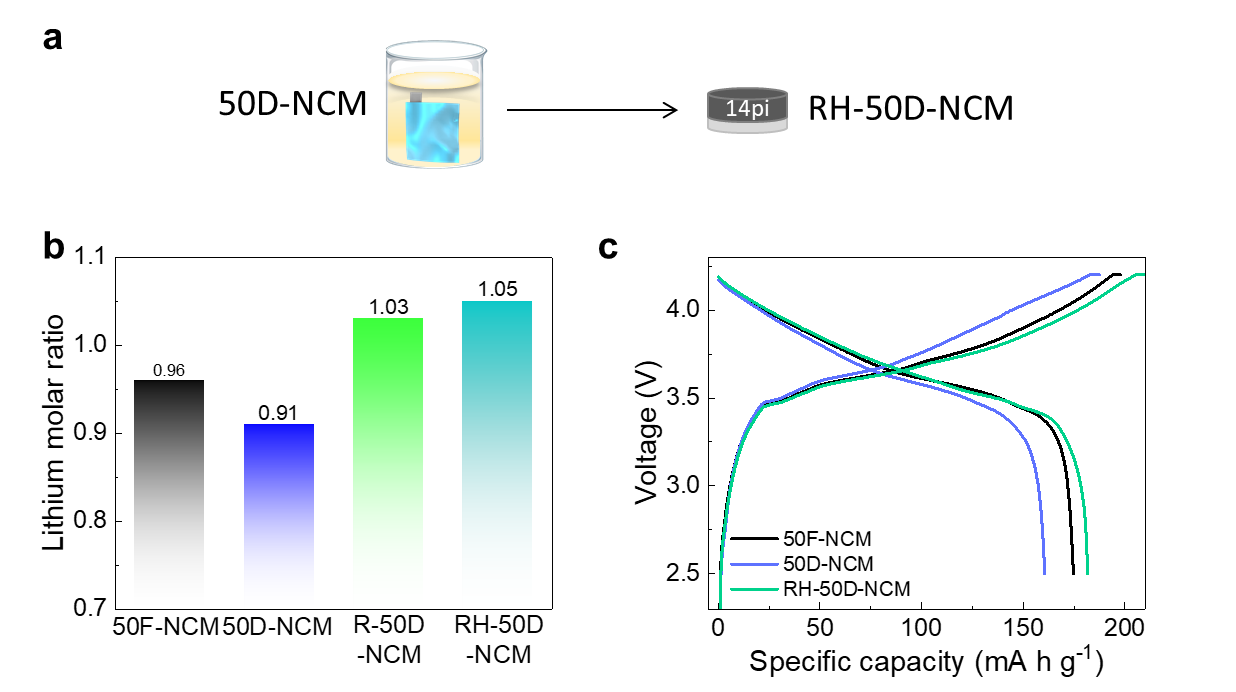


Figure S29 Relithiation results of 50D-NCM: (a) experimental scheme, (b) ICP-OES, and (c) voltage profile.

Re1-solution


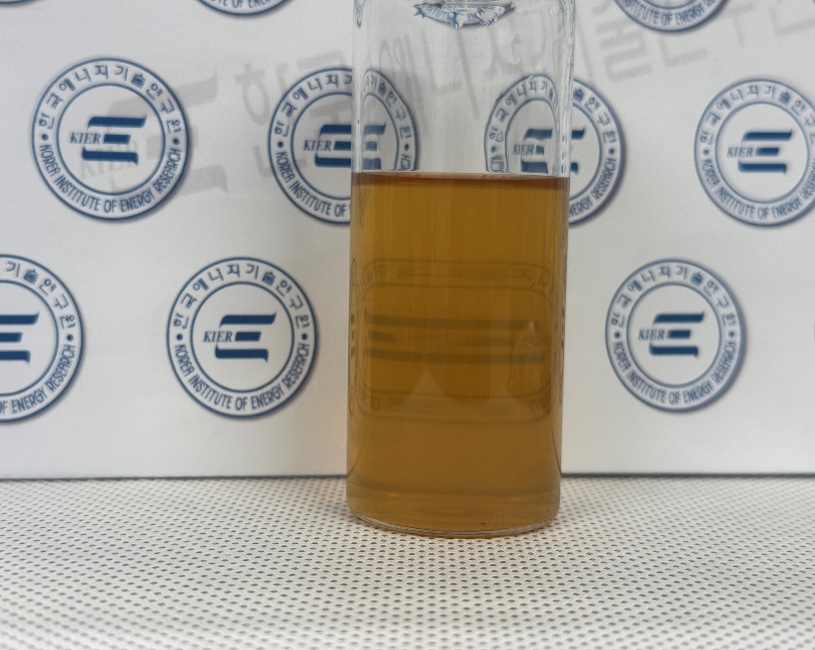


Re3-solution


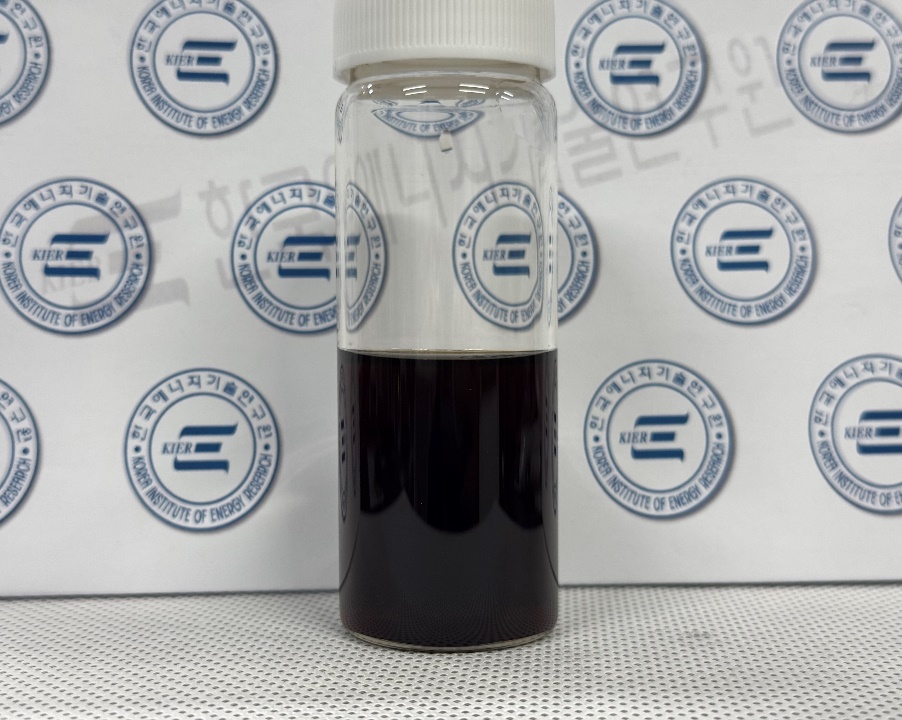

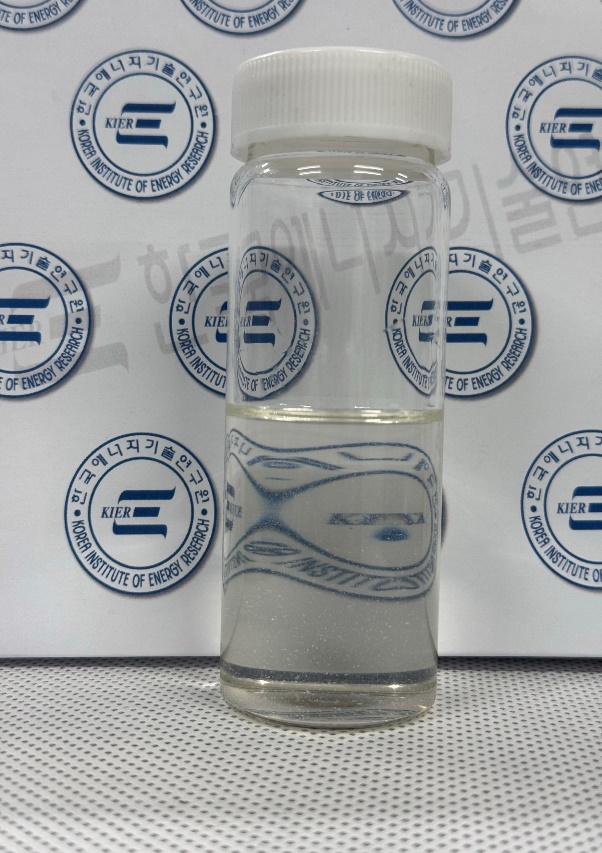


Solution

Figure S30 Reaction solution images of the polyol solution: fresh, after one reaction cycle, and after three consecutive reaction cycles (subsequently steps showed similar colors).


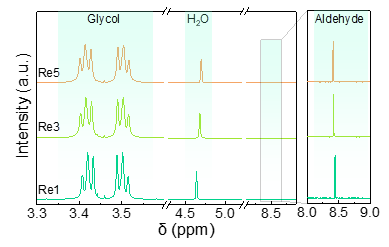


Figure S31 1H-NMR results of polyol solution after repeated reactions.

Table S8 1H-NMR integral results for polyol solution after repeating the reaction one, three, and five times: Re1, Re3, and Re5.


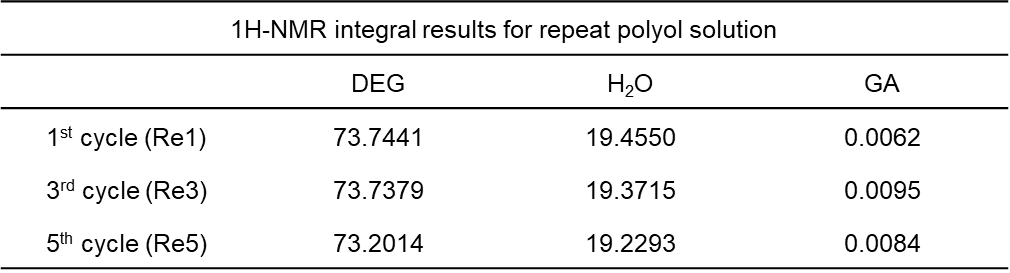


Table S9 Detailed numerical data data from Figure 5b and the setting process for each recovery method used in EverBatt.


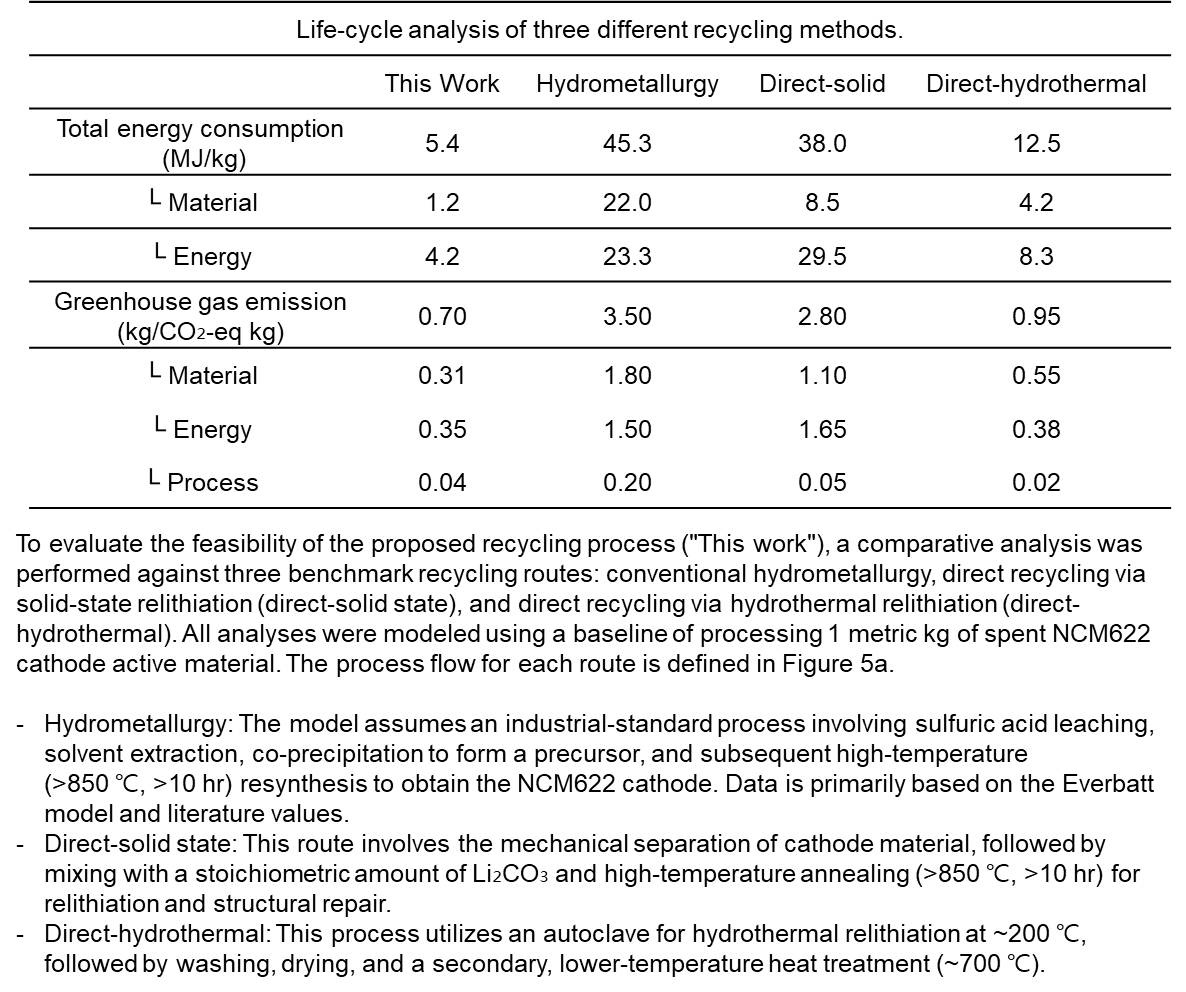


Table S10 Main materials used in different recycling processes.


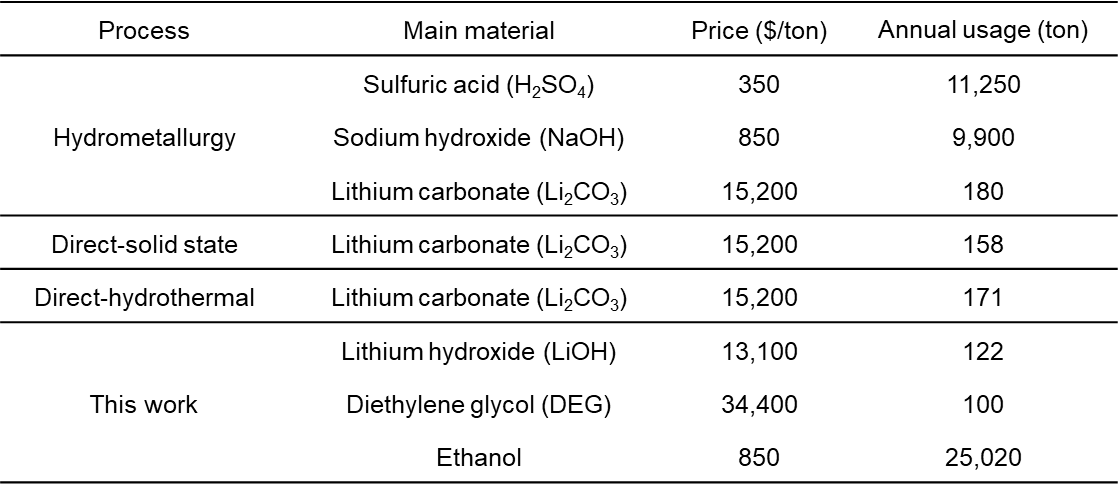


Table S11 Main products from different recycling processes.


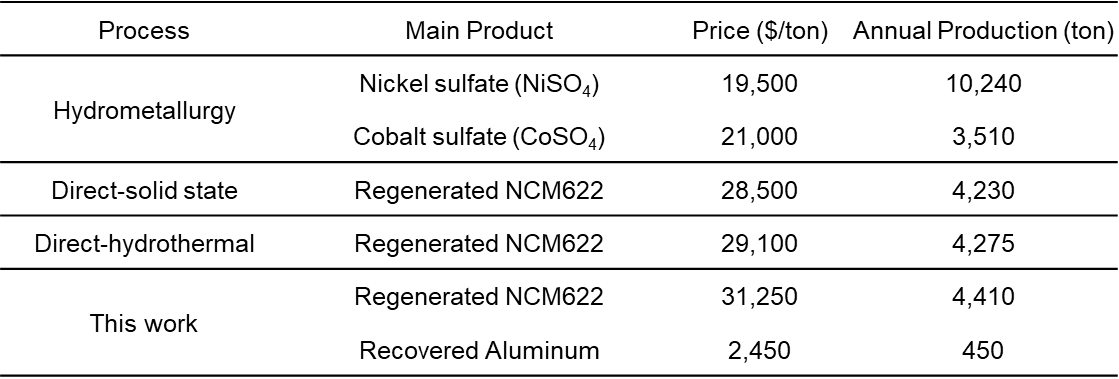


Table S12 Energy consumption of different recycling processes.


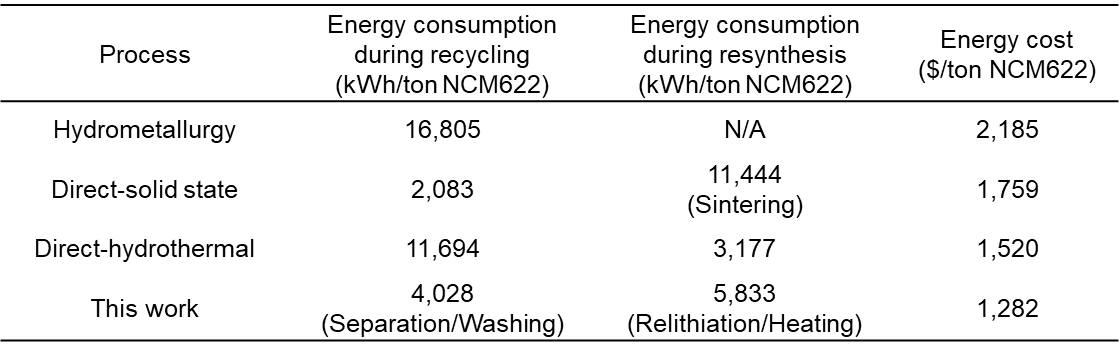


Table S13 Specific components of CO₂ emission estimations.


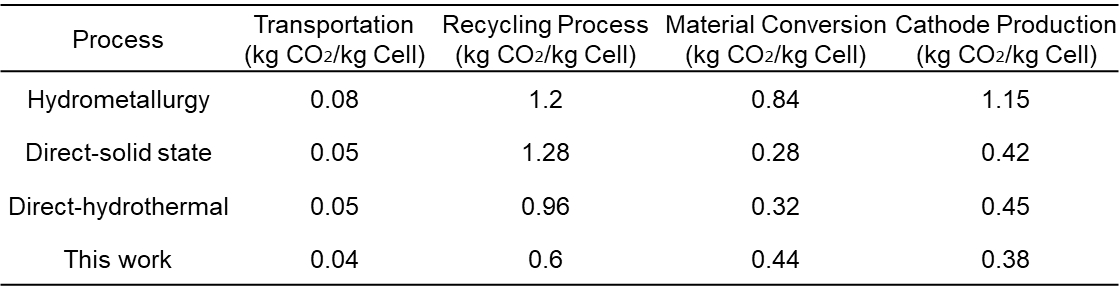


Table S14 Environmental protection fees of different recycling processes.


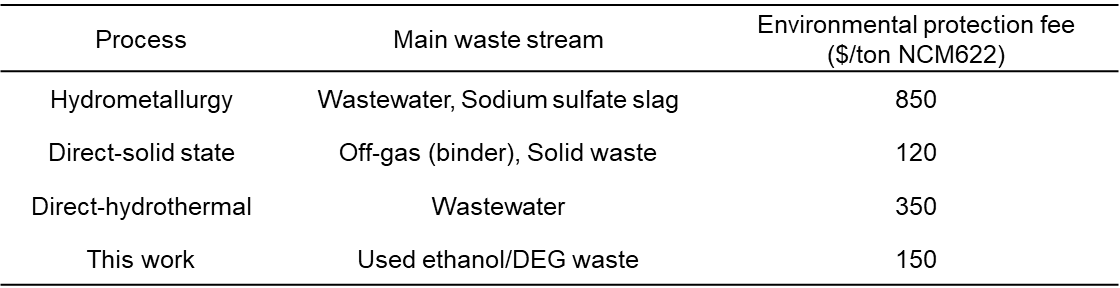


Table S15 Production cost of different recycling processes.


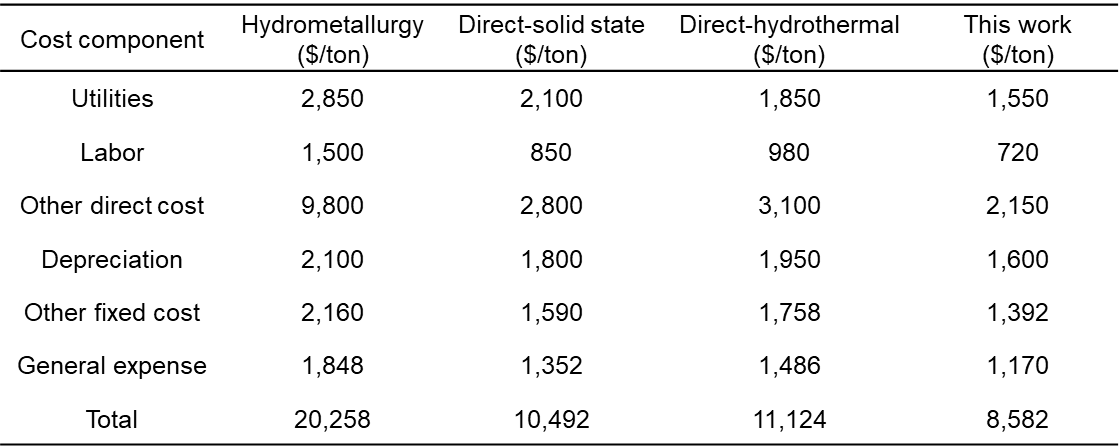

Supplement: Supplementary file 1 — Supporting File: advs75879‐sup‐0001‐SuppMat.docx. [file ADVS-9999-e75879-s001.docx]
